# Socioeconomic inequalities and dynamic changes in sex differences in lifetime risks of peptic ulcer disease

**DOI:** 10.1186/s13293-026-00832-w

**Published:** 2026-01-27

**Authors:** Dequan Shi, Yuhao Li, Rongshou Zheng, Shengfeng Wang, Ru Chen

**Affiliations:** 1 National Central Cancer Registry, National Cancer Center, National Clinical Research Center for Cancer/Cancer Hospital, Chinese Academy of Medical Sciences and Peking Union Medical College, Beijing, China; 2 Department of Epidemiology and Biostatistics, School of Public Health, Peking University, Beijing, China; 3 Key Laboratory of Epidemiology of Major Diseases (Peking University), Ministry of Education, Haidian District, Beijing, China; 4 Institute for Artificial Intelligence, Peking University, Beijing, China

**Keywords:** Global, Peptic ulcer disease, Lifetime risk, Burden, Sex difference

## Abstract

**Background:**

Peptic ulcer disease (PUD) is a common digestive system disorder and an important risk factor for gastric cancer. While previous studies have extensively focused on using traditional indicators, lifetime risks of PUD remain relatively scarce.

**Methods:**

Using Global Burden of Disease (GBD) 2021 data, we estimated lifetime risks of developing and dying from PUD by lifetable method.Trends were assessed by calculating the average annual percent change (AAPC) from 1990 to 2021. By computing the sex ratios(male to female) of lifetime risks and plotting time-trend graphs, we analyzed the dynamic evolution of sex differences.

**Results:**

In 2021, the global lifetime risk of developing from PUD was 3.21% (95% CI, 3.20%-3.22%), declining from 1990 (AAPC: -1.24; 95% CI, -1.37 to -1.11), with a more pronounced decrease among males (AAPC: -1.43; 95% CI, -1.53 to -1.33) than females (AAPC: -1.00; 95% CI, -1.10 to -0.89). The lifetime risk of dying from PUD was 0.35% (95% CI, 0.34%-0.35%), with a faster decline (AAPC, -2.25; 95% CI, -2.57 to -1.93), again with greater in males (AAPC: -2.73; 95% CI, -2.86 to -2.60) than in females (AAPC: -1.80; 95% CI, -2.00 to -1.60). Marked socioeconomic disparities were observed: high-SDI regions had the highest lifetime risk of developing but the lowest risk of dying, whereas low-SDI regions showed the opposite pattern. Across different SDI regions, the sex ratios of lifetime risk of PUD exhibited unique inflection points over three decades.

**Conclusion:**

Despite substantial global declines in lifetime risks of PUD over the past three decades, our findings reveal persistent inequities by SDI, geography, and sex. These disparities underscore that access to timely diagnosis, eradication therapy, and advanced endoscopic care remains uneven, particularly in low-SDI regions and among females.

**Supplementary Information:**

The online version contains supplementary material available at 10.1186/s13293-026-00832-w.

## Highlights

This study presents the first comprehensive assessment of lifetime risks of developing and dying from peptic ulcer disease (PUD) across 21 GBD regions and 5 sociodemographic index (SDI) areas, revealing significant disparities, not only between different SDI regions but also between males and females. We also identified an important trend diverging from previous studies, a fundamental reversal in lifetime risks of PUD between the sexes. Specifically, for lifetime risk of developing PUD, the inflection point at which females risk surpassed that of males occurred in 1998 in low-middle SDI regions, 2013 in low-SDI regions, and 2019 in high-SDI regions. For lifetime risk of dying from PUD, the corresponding inflection points occurred in 2006 in low-middle SDI regions, 2012 in middle and low-SDI regions , and 2020 globally.

**Supplementary Information:**

The online version contains supplementary material available at 10.1186/s13293-026-00832-w.

## Abstract (plain language summary)

Our study investigated a person’s lifetime probability of developing or dying from a peptic ulcer. Using the most comprehensive global health data from 1990 to 2021, we calculated this “lifetime risk” for males and females across countries with varying development levels, with a specific focus on analyzing differences between the sexes.

Firstly, from a total population perspective, the lifetime risk of anyone developing or dying from an ulcer has significantly declined since 1990. Secondly, the differences between men and women have undergone dynamic changes over these three decades. While previous research consistently indicated a higher risk for men, our study reveals that this long-standing pattern has reversed. We identified “tipping points” after which women’s lifetime risk of peptic ulcers surpassed that of men. This reversal has already occurred in poorer regions and is now emerging in the wealthiest nations.

Our findings challenge the conventional belief that peptic ulcer disease is a “male disease.” The biology of this condition is closely intertwined with societal factors, and the shift in risk towards women is particularly pronounced in low-income regions. This research sounds an alarm: we can no longer overlook the increasing burden of peptic ulcers among women, and future efforts must prioritize addressing this risk in the female population.

**Supplementary Information:**

The online version contains supplementary material available at 10.1186/s13293-026-00832-w.

## Introduction

Peptic ulcer disease (PUD) is a common upper gastrointestinal disorder that primarily affects the stomach and proximal duodenum. Its pathogenesis stems from an imbalance between gastric mucosal defense mechanisms and the damaging effects of gastric acid and pepsin [1–3]. Impairment of mucosal defenses, combined with gastric acid hypersecretion, can lead to progression from superficial mucosal injury to deep ulceration, which manifests as symptoms such as abdominal pain and dyspepsia [4]. Acute complications, including gastrointestinal bleeding or perforation, may lead to life-threatening conditions such as shock and sepsis [5, 6]. Moreover, PUD is associated with an increased risk of gastric cancer [7]. In 2021, there were an estimated 2.85 million new cases of PUD and over 230,000 related deaths worldwide [8]. The disease considerably impairs quality of life, and the associated costs of diagnosis, treatment, and hospitalization pose significant challenges to global public health [9–11].

The lifetime risk of PUD represents the probability that an individual will develop or die from it between birth and death, serving as a crucial longitudinal measure of disease burden [12, 13]. Compared with conventional metrics such as incidence, mortality, or cumulative risk from ages 0 to 74 years, lifetime risk incorporates life expectancy, the potential for multiple occurrences, and competing risks from other causes of death, thereby providing a more intuitive perspective on disease burden [12, 13]. Previous studies have identified Helicobacter pylori (H.pylori) infection and nonsteroidal anti-inflammatory drugs (NSAIDs) use as the most common etiologic factors for PUD [14, 15]. The burden of PUD is closely linked to socioeconomic factors and health care accessibility, with the highest age-standardized prevalence rates, mortality rates, and disability-adjusted life-years (DALYs) observed in low- and low-middle sociodemographic index (SDI) regions [16–21].

Sex-specific analyses indicated a consistently higher burden among males than among females from 1990 to 2021 [19, 22]. This disparity may be attributed to physiological and behavioral factors, males typically exhibit higher basal gastric acid secretion and are more likely to engage in risk behaviors such as smoking and alcohol consumption. In contrast, premenopausal women may benefit from the protective effects of estrogen, which enhances mucosal defense through multiple mechanisms including inhibition of acid secretion and stimulation of prostaglandin synthesis. However, this sex-based difference may not be static but rather exhibits dynamic patterns across the lifespan and socioeconomic strata. The protective advantage in women appears to diminish after menopause due to declining estrogen levels, while factors such as nutritional deficiencies in low-SDI regions and increased NSAID use in high-SDI regions may further modulate sex-specific disease risks over time [23, 24].

Leveraging data from the 2021 Global Burden of Disease (GBD) study, this study aimed to estimate the lifetime risks of developing and dying from PUD at global, regional, and SDI-stratified levels. By examining trends from 1990 to 2021, assessing geographic disparities, and evaluating dynamic sex-specific differences, this study seeks to provide a scientific foundation for understanding the burden of PUD and informing global health policies.

## Methods

### Data sources

The GBD 2021 study is a comprehensive epidemiological assessment that provides data on 371 diseases and injuries worldwide [8]. Data sources included vital registration systems, epidemiological surveys, disease surveillance systems, cancer registries, police records, and open-source databases. Detailed methods for data collection and processing have been described previously. Downstream analyses used advanced statistical models, including meta-regression with Bayesian priors, regularization, and trimming (MR-BRT); DisMod-MR 2.1; and spatiotemporal Gaussian process regression (ST-GPR). All disease terms were standardized using International Classification of Diseases (ICD) codes to ensure accuracy and comparability. The study adhered to the Guidelines for Accurate and Transparent Health Estimates Reporting (GATHER). We extracted age-, sex-, and location-specific incidence and mortality rates for PUD, from 1990 to 2021, as well as total population and all-cause mortality rates. Data encompassed all 21 GBD regions. In addition, we used the SDI, a composite indicator of development level calculated as the geometric mean of 3 scaled components: total fertility rate among persons younger than 25 years, mean educational attainment among persons 15 years or older, and lag-distributed income per capita. The SDI ranges from 0 (lowest development) to 1 (highest development). Based on SDI values, countries and territories were categorized into 5 quintiles: low, low-middle, middle, high-middle, and high SDI, representing varying socioeconomic levels.

### Statistical analysis

We estimated lifetime risks of developing and dying from PUD for the overall population, males, and females across 21 regions, and 5 SDI levels using the lifetable method(Additional file1; Supplementary File 2) [12, 13].This approach assumes a PUD related mortality risk of 0 and corrects for the effects of multiple primary events in incidence rates. Age-specific incidence, mortality, and all-cause mortality rates, stratified by 5-year age groups, were used to calculate lifetime risks for males and females in each age group, representing the probability of developing or dying from PUD from the index age onward. Lifetime risks were assumed to be uniformly distributed within the given 95% CI, and male-to-female lifetime risk ratios with 95% CIs were derived via 100 000 resamples.

**Table 1:** Lifetime risks(%) of developing and dying from PUD in 1990 and 2021, both sex

**Location**	**LRDE (95% CI)**			**AAPC(95% CI)**,**1990–2021**	**P-valuefor AAPC **	**LRDY (95% CI)**		**AAPC(95% CI)**,**1990–2021**	**P-value for AAPC**
	**1990**	**2021**				**1990**	**2021**		
Global	4.70(4.69,4.71)	3.21(3.20,3.22)		−1.24 (−1.37, −1.11)	< 0.001	0.70(0.70,0.70)	0.35(0.34,0.35)	−2.25 (−2.57, −1.93)	< 0.001
High SDI	4.76(4.73,4.79)	3.85(3.81,3.88)		−0.69 (−0.76, −0.62)	< 0.001	0.53(0.52,0.53)	0.23(0.22,0.23)	−2.71 (−3.04, −2.38)	< 0.001
High-middle SDI	4.05(4.03,4.07)	2.89(2.87,2.92)		−1.07 (−1.21, −0.92)	< 0.001	0.46(0.45,0.46)	0.28(0.28,0.29)	−1.54 (−1.79, −1.29)	< 0.001
Middle SDI	5.22(5.20,5.23)	3.14(3.13,3.15)		−1.62 (−1.77, −1.48)	< 0.001	0.77(0.76,0.77)	0.35(0.35,0.35)	−2.53 (−2.70, −2.35)	< 0.001
Low-middle SDI	5.45(5.43,5.47)	3.20(3.18,3.21)		−1.71 (−1.83, −1.60)	< 0.001	1.12(1.11,1.13)	0.47(0.47,0.48)	−2.77 (−3.06, −2.48)	< 0.001
Low SDI	3.66(3.64,3.68)	2.75(2.74,2.77)		−0.96 (−1.14, −0.77)	< 0.001	0.95(0.94,0.96)	0.50(0.50,0.51)	−2.10 (−2.41, −1.78)	< 0.001
Regional									
Southern Latin America	1.86(1.82,1.91)	1.48(1.44,1.53)		−0.75 (−0.87, −0.63)	< 0.001	0.37(0.35,0.40)	0.17(0.16,0.18)	−2.44 (−3.29, −1.59)	< 0.001
Western Europe	2.81(2.79,2.83)	1.86(1.83,1.89)		−1.37 (−1.43, −1.31)	< 0.001	0.57(0.56,0.57)	0.21(0.21,0.21)	−3.21 (−3.38, −3.05)	< 0.001
High-income North America	5.85(5.81,5.90)	4.76(4.71,4.80)		−0.70 (−0.77, −0.63)	< 0.001	0.34(0.33,0.35)	0.12(0.12,0.13)	−3.38 (−4.04, −2.73)	< 0.001
Australasia	3.96(3.84,4.08)	2.00(1.89,2.11)		−2.19 (−2.30, −2.07)	< 0.001	0.79(0.74,0.84)	0.19(0.17,0.21)	−4.41 (−5.04, −3.77)	< 0.001
High-income Asia Pacific	5.97(5.87,6.07)	4.78(4.57,5.00)		−0.71 (−0.90, −0.52)	< 0.001	0.64(0.63,0.66)	0.24(0.23,0.25)	−3.12 (−3.52, −2.72)	< 0.001
Caribbean	2.94(2.83,3.05)	2.11(2.02,2.20)		−1.12 (−1.52, −0.71)	< 0.001	0.74(0.70,0.78)	0.34(0.32,0.35)	−2.14 (−2.44, −1.85)	< 0.001
Central Latin America	3.26(3.22,3.31)	1.66(1.64,1.68)		−2.26 (−2.43, −2.10)	< 0.001	1.16(1.13,1.19)	0.35(0.34,0.36)	−3.95 (−4.36, −3.55)	< 0.001
Tropical Latin America	3.71(3.66,3.75)	1.81(1.79,1.83)		−2.28 (−2.55, −2.00)	< 0.001	0.52(0.50,0.54)	0.28(0.27,0.29)	−1.94 (−2.30, −1.58)	< 0.001
Andean Latin America	3.59(3.48,3.71)	1.89(1.84,1.94)		−2.11 (−2.32, −1.91)	< 0.001	1.04(0.98,1.10)	0.22(0.20,0.23)	−5.27 (−5.86, −4.67)	< 0.001
Central Sub-Saharan Africa	2.30(2.25,2.35)	2.30(2.25,2.34)		−0.02 (−0.17, 0.12)	0.761	0.54(0.52,0.57)	0.42(0.41,0.44)	−0.89 (−1.08, −0.69)	< 0.001
Eastern Sub-Saharan Africa	2.14(2.12,2.17)	1.97(1.95,1.99)		−0.36 (−0.70, −0.02)	< 0.001	0.72(0.71,0.74)	0.48(0.47,0.48)	−1.32 (−1.87, −0.77)	< 0.001
Southern Sub-Saharan Africa	2.70(2.64,2.77)	1.84(1.81,1.87)		−1.22 (−1.57, −0.88)	< 0.001	0.57(0.54,0.60)	0.26(0.25,0.27)	−2.57 (−2.94, −2.21)	< 0.001
Western Sub-Saharan Africa	2.93(2.89,2.97)	2.94(2.91,2.98)		−0.01 (−0.10, 0.08)	0.812	0.57(0.56,0.59)	0.43(0.42,0.43)	−0.91 (−1.08, −0.75)	< 0.001
North Africa and Middle East	3.08(3.05,3.11)	2.41(2.39,2.44)		−0.81 (−0.89, −0.73)	< 0.001	0.56(0.54,0.57)	0.21(0.21,0.22)	−3.17 (−3.35, −2.99)	< 0.001
South Asia	6.33(6.31,6.36)	3.30(3.28,3.32)		−2.08 (−2.22, −1.94)	< 0.001	1.31(1.30,1.32)	0.51(0.50,0.51)	−3.02 (−3.30, −2.74)	< 0.001
East Asia	6.32(6.29,6.34)	3.89(3.87,3.92)		−1.57 (−1.68, −1.46)	< 0.001	0.74(0.74,0.75)	0.35(0.34,0.35)	−2.41 (−2.63, −2.20)	< 0.001
Southeast Asia	4.36(4.33,4.39)	3.16(3.13,3.19)		−1.03 (−1.23, −0.82)	< 0.001	0.70(0.69,0.71)	0.39(0.39,0.40)	−1.76 (−1.97, −1.54)	< 0.001
Oceania(Expect Australasia)	5.66(5.29,6.02)	4.60(4.35,4.86)		−0.63 (−0.80, −0.45)	< 0.001	0.83(0.72,0.94)	0.45(0.40,0.50)	−1.85 (−2.04, −1.65)	< 0.001
Central Asia	2.79(2.72,2.85)	2.75(2.68,2.81)		−0.07 (−0.29, 0.15)	0.526	0.38(0.36,0.40)	0.34(0.33,0.36)	−0.45 (−0.88, −0.01)	< 0.001
Eastern Europe	3.06(3.03,3.09)	3.08(3.04,3.13)		0.01 (−0.38, 0.40)	0.958	0.33(0.32,0.33)	0.42(0.41,0.42)	0.79 (−0.21, 1.80)	0.121
Central Europe	3.88(3.83,3.94)	3.59(3.49,3.69)		−0.23 (−0.35, −0.11)	< 0.001	0.51(0.49,0.52)	0.44(0.43,0.45)	−0.47 (−0.90, −0.04)	< 0.001

LRDE = lifetime risk of developing(%); LRDY = lifetime risk of dying(%);CI = confidence interval; SDI: sociodemographic index

This study assessed temporal trends in lifetime risk using the average annual percentage change (AAPC) with corresponding 95% confidence intervals (CIs), which were calculated with the Joinpoint Trend Analysis Software (command line version 5.3.0)(Supplementary File 2). The AAPC serves as a summary measure that captures the average rate of change over a specified period, while accounting for potential shifts in trend direction. Joinpoint regression analysis identifies points in time where statistically significant changes in trends occur by fitting a sequence of joined straight lines on a logarithmic scale. The software applies a grid search method to determine the optimal number and location of joinpoints, and assesses statistical significance using Monte Carlo permutation tests. This approach enables the detection of complex temporal patterns and provides robust estimates of trend changes, making it particularly suitable for analyzing long-term epidemiological data characterized by potential dynamic variations over time [25]. Negative AAPC indicates a declining trend, while positive AAPC indicates an increasingtrend.

We calculated time-varying sex ratios (male to female) for lifetime risks of developing and dying from PUD across SDI levels and identified inflection points (the point after which female lifetime risks exceeded those of males). We assessed the strength and direction of correlations between lifetime risk of developing PUD and cumulative risks from ages 0 to 74 years using standardized linear regression models and computed differences between estimates with 95% CIs. Standardized linear regression model refers to a quadratic regression model wherein the predictor variable (cumulative risk from 0 to 74 years) was standardized prior to model fitting. The cumulative risk values were initially transformed into Z-scores; subsequently, both these standardized values and their squared terms were incorporated into the model to establish the quadratic regression framework.The 95% CIs were calculated assuming a Poisson distribution for lifetime risk of developing and dying from PUD. All analyses were performed using SAS (version 9.4) and R (version 4.4.1).

## Results

### Global and regional lifetime risks of developing and dying from PUD

#### Lifetime risk of developing from PUD

**Fig. 1:**
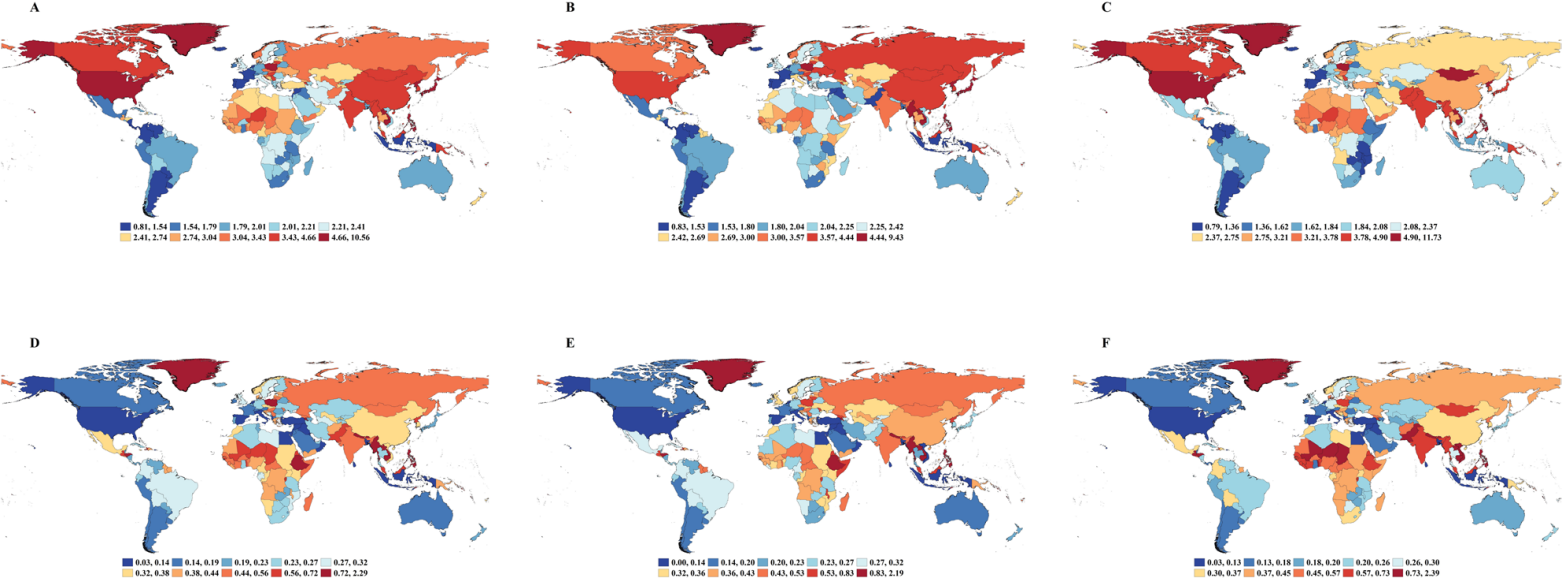
Lifetime risks of developing and dying from PUD for the whole population, males and females in 2021. **A** Lifetime risk of developing PUD in the whole population **B** Lifetime risk of developing PUD in males **C** Lifetime risk of developing PUD in females **D** Lifetime risk of dying from PUD in the whole population **E** Lifetime risk of dying from PUD in males **F** Lifetime risk of dying from PUD in females

**Fig. 2:**
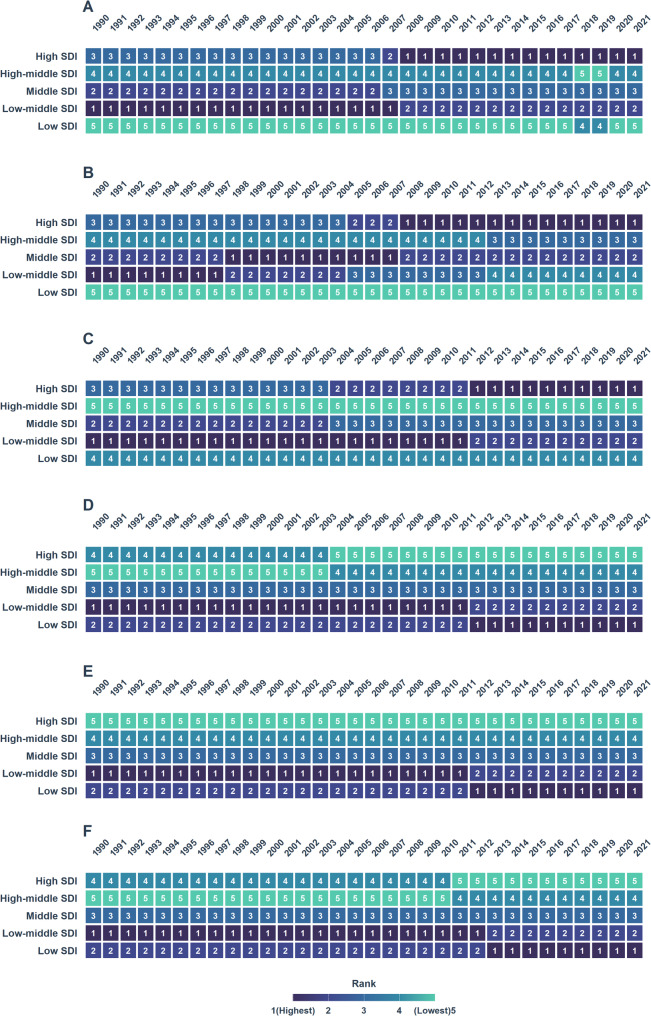
Heatmap of the lifetime risk of developing and dying from PUD by SDI for the whole population, males and females from 1990 to 2021 **A** Lifetime risk of developing PUD in the whole population **B** Lifetime risk of developing PUD in males **C** Lifetime risk of developing PUD in females **D** Lifetime risk of dying from PUD in the whole population **E** Lifetime risk of dying from PUD in males **F** Lifetime risk of dying from PUD in females

**Fig. 3:**
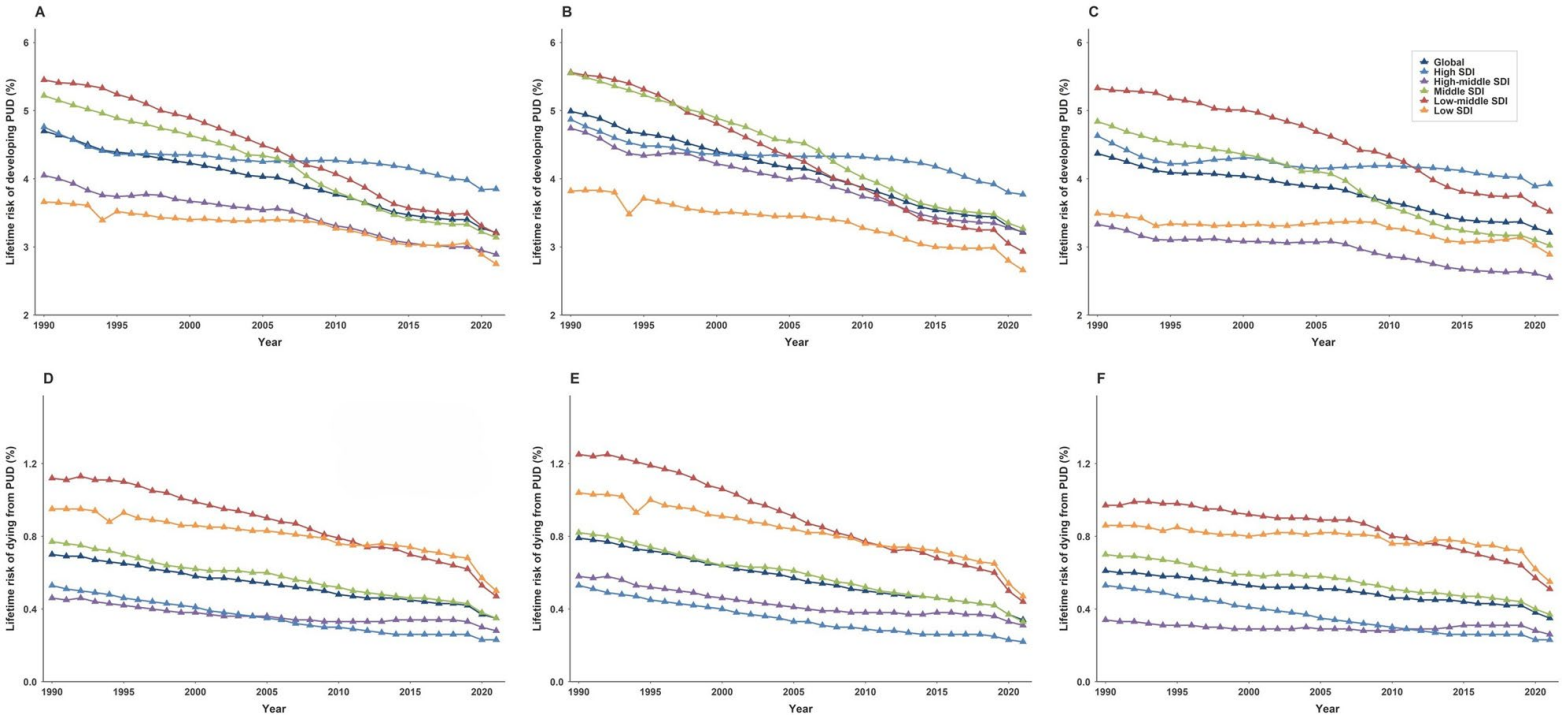
Trends in lifetime risks of developing and dying from PUD by SDI for the whole population, males and females from 1990 to 2021 **A** Trends in lifetime risk of developing from PUD in the whole population **B** Trends in lifetime risk of developing from PUD in males **C** Trends in lifetime risk of developing from PUD in females **D** Trends in lifetime risk of dying from PUD in the whole population. **E** Trends in lifetime risk of dying from PUD in males **F** Trends in lifetime risk of dying from PUD in females

The global lifetime risk of developing from PUD was 3.21% in 2021 (95% CI, 3.20%−3.22%) (Table 1; Fig. 1A).Although this risk was nearly consistent for both males (3.21%; 95% CI, 3.20%−3.22%) and females (3.21%; 95% CI, 3.20%−3.23%), significant regional variations were observed (Table 1; Fig. 1A, Additional file1:Table S1, Table S2). High-income Asia Pacific exhibited the highest lifetime risk of developing (4.78%; 95% CI, 4.57%−5.00%), while Southern Latin America had the lowest risk (1.86%; 95% CI, 1.82%−1.91%) (Table 1; Fig. 1A, Additional file1:Table S1, Table S2, Fig.S1A). In 2021, significant disparities in lifetime risk of developing from PUD were also observed across SDI regions. The highest risk was in high-SDI regions (3.85%; 95% CI, 3.81%−3.88%) and the lowest in low-SDI regions (2.75%; 95% CI, 2.74%−2.77%)(Table 1; Fig. 2A).Globally, the lifetime risk of developing PUD in 2021 demonstrated a significant decreasing trend compared to 1990, with an AAPC of − 1.24 (95% CI: −1.37 to − 1.11,*P* < 0.001). Males experienced a faster decline (AAPC:−1.43; 95% CI,−1.53 to − 1.33) than females (AAPC:−1.00; 95% CI,−1.10 to − 0.89,*P* < 0.001) (Table 1; Fig. 3A-C, Additional file1:Table S1,Table S2). The most rapid decline occurred in low-middle SDI regions (AAPC:−1.71; 95% CI, − 1.83 to − 1.60,*P* < 0.001), contrasting with the slowest reduction in high-SDI regions (AAPC, − 0.69; 95% CI, − 0.76 to − 0.62,*P* < 0.001) (Table 2, Additional file1:Fig.S2A, Fig.S3A). For males, the fastest decrease occurred in low-middle SDI regions (AAPC, − 2.06; 95% CI, − 2.19 to − 1.93,*P* < 0.001), while their risk in high-SDI regions showed the most modest decline (AAPC, − 0.82; 95% CI, − 0.91 to − 0.74,*P* < 0.001)(Table S1,Additional file1:Fig.S3B). Females exhibited the greatest improvement in middle-SDI regions (AAPC, − 1.51; 95% CI, − 1.68 to − 1.33,*P* < 0.001), with the high-SDI regions demonstrating the least progress (AAPC, − 0.54; 95% CI, − 0.66 to − 0.42,*P* < 0.001) (Table S2, Additional file1:Fig.S3C).

#### Lifetime risk of dying from PUD

The global lifetime risk of dying from PUD was estimated at 0.35% in 2021 (95% CI, 0.34%−0.35%), with a risk of 0.34% (95% CI, 0.34%−0.34%) for males and 0.35% (95% CI, 0.35%−0.36%) for females (Table 1; Fig. 1A, Additional file1:Table S1, Table S2). South Asia exhibited the highest lifetime risk of death (0.51%; 95% CI, 0.50%−0.51%), while High-Income North America had the lowest risk (0.12%; 95% CI, 0.12%−0.13%) (Table 1; Fig. 1D, Additional file1:Fig.S1D). In 2021, the highest lifetime risk of dying from PUD was found in low-SDI regions (0.50%; 95% CI, 0.50%−0.51%), while high-SDI regions showed the lowest risk (0.23%; 95% CI, 0.22%−0.23%) (Table 1; Fig. 2D).Globally, the AAPC in lifetime risk of dying from PUD showed a significant decline from 1990 to 2021, with an overall AAPC of − 2.25 (95% CI, − 2.57 to − 1.93,*P* < 0.001) (Table 1; Fig. 3D). Sex-specific analyses revealed a more pronounced decrease among males (AAPC: −2.73; 95% CI, − 2.86 to − 2.60,*P* < 0.001) compared with females (AAPC: −1.80; 95% CI, − 2.00 to − 1.60,*P* < 0.001) (Fig. 3E-F, Additional file1:Table S1, Table S2). From 1990 to 2021, low-middle SDI regions experienced the most rapid decline in lifetime risk of dying from PUD (AAPC: −2.77; 95% CI, − 3.06 to − 2.48,*P* < 0.001), whilehigh-middle SDI regions had the slowest reduction (AAPC: −1.54; 95% CI, − 1.79 to − 1.29,*P* < 0.001) (Table 1; Fig. 3D, Additional file1:Fig.S3D). For males, the fastest decrease occurred in low-middle SDI regions (AAPC: −3.31; 95% CI, − 3.52 to − 3.10,*P* < 0.001), whereas high-middle SDI regions showed the most modest decline (AAPC: −2.11; 95% CI, − 2.42 to − 1.80)(Fig. 3E, Additional file: Table S1, Fig.S3E). Among females, high-SDI regions exhibited the greatest improvement (AAPC: −2.69; 95% CI, − 2.91 to − 2.48), while high-middle SDI regions demonstrated the least progress (AAPC: −0.85; 95% CI, − 1.36 to − 0.35,*P* < 0.001) (Fig. 3F, Additional file: Table S2, Fig.S3F).

### Sex-specific disparities and Temporal trends in lifetime risks of developing and dying from PUD

#### Sex ratio in lifetime risks of developing from PUD

**Fig. 4:**
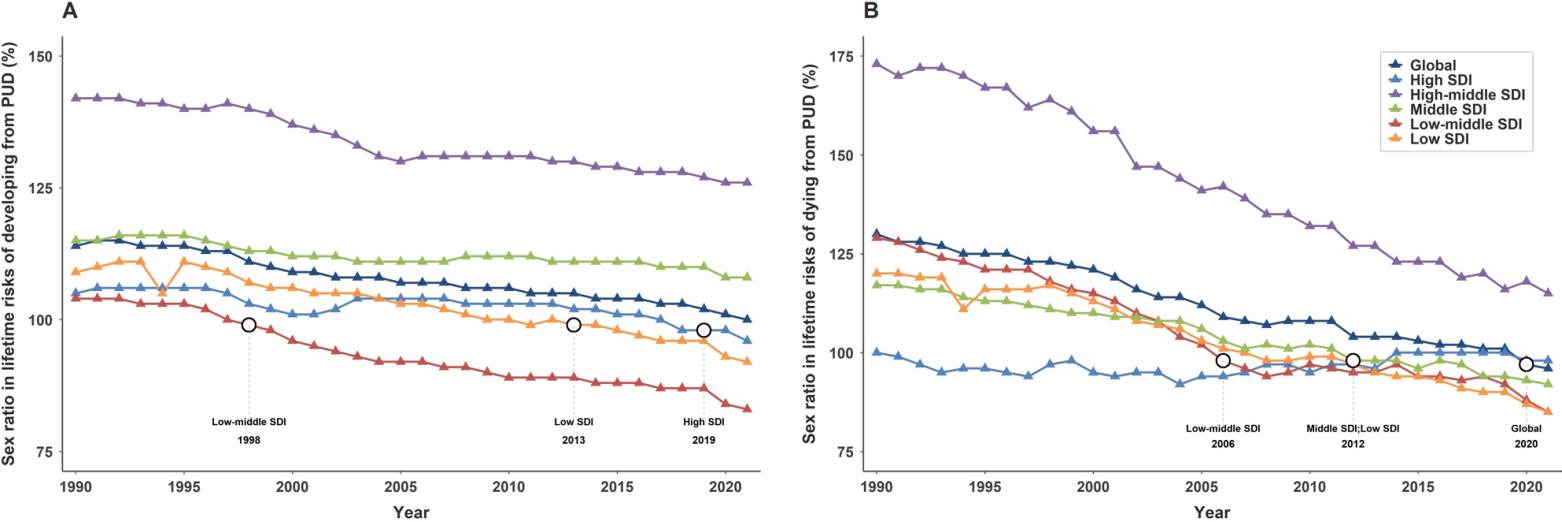
Sex ratio in lifetime risks of developing and dying from PUD by SDI from 1990 to 2021 **A** Sex ratio in lifetime risks of developing from PUD **B** Sex ratio in lifetime risks of dying from PUD

Globally, the sex ratio (male to female) in the lifetime risk of developing from PUD decreased from 1.14 (95% CI, 1.14–1.15) in 1990 to 1.00 (95% CI, 0.99–1.00.99.00) in 2021, approaching parity. Regional disparities were evident across SDI regions in 1990, with the highest male predominance in high-middle SDI regions (1.42; 95% CI, 1.41–1.44), followed by middle-SDI regions (1.15; 95% CI, 1.14–1.16), low-SDI regions (1.09; 95% CI, 1.08–1.11), high-SDI regions (1.05; 95% CI, 1.04–1.07), and low-middle SDI regions (1.04; 95% CI, 1.03–1.05) (Table 2). By 2021, all regions showed a decline in the sex ratio compared with 1990; low-middle SDI regions had the lowest ratio (0.83; 95% CI, 0.82–0.84), followed by low-SDI regions (0.92; 95% CI, 0.91–0.93), high-SDI regions (0.96; 95% CI, 0.94–0.99), middle-SDI regions (1.08; 95% CI, 1.07–1.09), and high-middle SDI regions (1.26; 95% CI, 1.24–1.29) (Table 2).The global AAPC in the sex ratio was − 0.42 (95% CI, − 0.45 to − 0.40,*P* < 0.001). The steepest decline occurred in low-middle SDI regions (AAPC: −0.71; 95% CI, − 0.72 to − 0.66,*P* < 0.001), while the smallest decline was observed in middle-SDI regions (AAPC: −0.19; 95% CI, − 0.22 to − 0.15,*P* < 0.001) (Table 2; Fig. 4A). Notably, by 2021, sex ratios in high-SDI, low-middle–SDI, and low-SDI regions had all fallen below 1.00, indicating a higher lifetime risk of developing PUD in females compared with males. However, in high-middle and middle-SDI regions, males continued to bear a higher overall risk than females, though the disparity has been gradually diminishing.In low-middle SDI regions, the inflection point(The year when the risk for females first exceeded that for males) occurred in 1998, after which the lifetime risk for females surpassed that of males. For low-SDI regions, the inflection point emerged in 2013, and in high-SDI regions, it appeared in 2019. (Fig. 4A, Additional file1:Table S3).

**Table 2:** The sex ratio of global and regional lifetime risk of developing and dying PUD in 1990 and 2021

Location	Sex ratio of LRDE (95% CI)		AAPC(95 CI%),1990–2021	*P*-value for AAPC	Sex ratio of LRDY (95% CI)			AAPC(95 CI%),1990–2021	*P*-value for AAPC
	1990	2021			1990		2021		
Global	1.14(1.14,1.15)	1.00(0.99,1.00)	−0.42(−0.45,−0.40)	< 0.001	1.30(1.28,1.31)	0.96(0.95,0.97)		−0.96(−1.01,−0.91)	< 0.001
High SDI	1.05(1.04,1.07)	0.96(0.94,0.99)	−0.23(−0.29,−0.17)	< 0.001	1.00(0.99,1.01)	0.98(0.96,1.00)		−0.18(−0.45, 0.09)	0.191
High-middle SDI	1.42(1.41,1.44)	1.26(1.24,1.29)	−0.42(−0.46,−0.38)	< 0.001	1.73(1.69,1.77)	1.15(1.12,1.18)		−1.44(−1.51,−1.38)	< 0.001
Middle SDI	1.15(1.14,1.16)	1.08(1.07,1.09)	−0.19(−0.22,−0.15)	< 0.001	1.17(1.15,1.20)	0.92(0.90,0.94)		−0.79(−0.83,−0.75)	< 0.001
Low-middle SDI	1.04(1.03,1.05)	0.83(0.82,0.84)	−0.71(−0.77,−0.66)	< 0.001	1.29(1.27,1.31)	0.85(0.85,0.86)		−1.29(−1.41,−1.16)	< 0.001
Low SDI	1.09(1.08,1.11)	0.92(0.91,0.93)	−0.55(−0.60,−0.50)	< 0.001	1.20(1.17,1.23)	0.85(0.83,0.88)		−1.10(−1.18,−1.03)	< 0.001

LRDE = lifetime risk of developing(%); LRDY = lifetime risk of dying(%);CI = confidence interval; SDI: sociodemographic index

#### Sex ratio in lifetime risks of dying from PUD

The global sex ratio for the lifetime risk of dying from PUD declined from 1.30 (95% CI, 1.28–1.31) in 1990 to 0.96 (95% CI, 0.95–0.97) in 2021. In 1990, high-middle SDI regions exhibited the largest male predominance (1.73; 95% CI, 1.69–1.77), followed by low-middle SDI regions (1.29; 95% CI, 1.27–1.31), low-SDI regions (1.20; 95% CI, 1.17–1.23), middle-SDI regions (1.17; 95% CI, 1.15–1.18).While high-SDI regions showed no sex disparity (1.00; 95% CI, 0.99–1.01) (Table 2). By 2021, sex ratios showed declining trends across all SDI regions except for high-SDI regions, which demonstrated no significant change. High-middle SDI regions (1.15; 95% CI, 1.12–1.18), middle-SDI regions (0.92; 95% CI, 0.90–0.94), low-middle SDI regions (0.85; 95% CI, 0.85–0.86), and low-SDI regions (0.85; 95% CI, 0.83–0.88).The most rapid decline occurred in high-middle SDI regions (AAPC:−1.44; 95% CI, − 1.51 to − 1.38,*P* < 0.001), while the slowest reduction was observed in middle-SDI regions (AAPC: −0.79; 95% CI, − 0.83 to − 0.75,*P* < 0.001). By 2021, with the exception of high-middle SDI regions where males continued to have a higher lifetime risk of dying than females, all other SDI regions demonstrated a higher lifetime risk of dying among females (Table 2; Fig. 4B). For lifetime risk of dying from PUD, the corresponding inflection points occurred in 2006 in low-middle SDI regions, 2012 in middle and low SDI regions. (Table 2; Fig. 4B, Additional file1:Table S4).

### Lifetime risks of developing and dying from PUD according to age at diagnosis in 2021

The lifetime risks of developing and dying from PUD decreased significantly with increasing age globally. For the birth-to-death interval, the overall lifetime risk of developing PUD was 3.21% (95% CI, 3.20%−3.22%), which declined to 1.10% (95% CI, 1.10%−1.10%) for those starting at age 70 years. Similarly, the lifetime risk of dying from PUD decreased from 0.35% (95% CI, 0.34%−0.35%) to 0.23% (95% CI, 0.23%−0.23%) across the same age intervals, with a more pronounced reduction observed after age 50 years(Additional file1:Table 5 and Fig.S4).

### Comparison between lifetime risk of developing PUD and cumulative risk for ages 0 to 74 years

In 2021, the global cumulative risk of PUD was 3.64% (95% CI, 3.62%−3.66%), higher than the lifetime risk of developing PUD (3.21%; 95% CI, 3.20%−3.22%). Significant variations were observed across SDI regions, the difference between lifetime risk of developing and cumulative incidence risk was greatest in low-SDI regions, reaching 1.37% (95% CI, 1.25%−1.49%). In contrast, in high-SDI regions, cumulative incidence risk was lower than the lifetime risk of developing, with a difference of − 0.45% (95% CI, − 0.55% to − 0.35%)(Additional file1:Table 6 and Fig.S5).

## Discussion

Using the GBD 2021 database, this study provides the first comprehensive assessment of lifetime risks of developing and dying from PUD across 21 GBD and 5 SDI regions. Our findings indicate significant global declines in both lifetime risks over the past three decades, alongside pronounced sex and geographic disparities. Previous studies have consistently documented substantial decreases in age-standardized incidence, prevalence, mortality, and disability-adjusted life years(DALYS) of PUD globally since 1990, with these studies consistently reporting higher disease burden in low and low-middle SDI regions and a generally greater burden among males than females. However, using lifetime risk as a new metric, we identified dynamic inflection points where females risks surpassed males risks, challenging prior assumptions of uniformly higher PUD burden in males [19–22, 26].

Between 1990 and 2021, global lifetime risk of developing from PUD decreased from 4.70% to 3.21%, and lifetime risk of dying declined from 0.70% to 0.35%, signifying an overall reduction in the global PUD burden [20–22, 26–28]. The reduction in lifetime risks may be attributed to global efforts in controlling H. pylori infection, rational antibiotic use [29–33], and widespread proton pump inhibitor (PPI) adoption [34–39]. The decline in lifetime risk of dying is likely closely linked to the extensive application of endoscopic technologies; improvements in early diagnosis and treatment have enabled timely and effective intervention for severe PUD complications [40–42]. It is noteworthy that the largest declines in lifetime risks of developing and dying occurred in low-middle SDI regions, likely reflecting higher baseline risks and accelerated improvements.

In 2021, lifetime risk distribution showed marked asymmetry across SDI levels: high-SDI regions carried the highest risk of developing PUD but the lowest risk of dying, while low-SDI regions exhibited the opposite pattern. This asymmetry phenomenon highlights inequities in health resource distribution. Factors contributing to the higher lifetime risk of developing in high-SDI regions include longer life expectancy, lower competing mortality risks from infectious diseases [43–45], and prevalent factors like diets high in fat and sugar [46], NSAIDs use in secondary prevention of cardiovascular and cerebrovascular disease [34], and psychological stressors such as anxiety and depression [47]. At a biological level, chronic psychological stress activates the hypothalamic-pituitary-adrenal axis and sympathetic nervous system, leading to catecholamine and corticosteroid release. This cascade induces intense gastric mucosal vasoconstriction mediated by angiotensin II and endothelin-1, while simultaneously suppressing key defensive mediators like prostaglandins and mucus secretion, thereby disrupting the mucosal barrier and increasing susceptibility to PUD [48]. However, standardized PPI use and widespread availability of advanced endoscopic hemostasis techniques significantly reduced lifetime risk of dying in these regions, while established gastrointestinal cancer screening systems in high-SDI regions concurrently enhanced early-stage PUD intervention rates [7, 39, 49]. In contrast, low-SDI regions face shorter life expectancy, higher competing mortality risks, low endoscopic screening rates, and nonstandardized diagnostic practices, leading to underdiagnosis and thus lower observed lifetime risk of developing [8, 50, 51]. Yet, due to nutritional deficiencies, scarce medical resources, inadequate H. pylori eradication, and limited treatment accessibility, patients with severe PUD often lack timely access to critical care, resulting in higher lifetime risk of dying compared with high-SDI regions [31, 32, 52]. The widespread nutritional deficiencies in these regions, particularly of zinc (a critical cofactor for DNA synthesis and cellular proliferation) and selenium, directly impair mucosal healing and repair processes. Concurrently, limited access to eradication therapy allows H. pylori-induced chronic inflammation and oxidative stress to persist unchecked, significantly increasing the risk of lethal complications [48, 53, 54].These findings suggest that high-SDI regions should further refine NSAIDs prescription management and promote healthy lifestyles, while low-SDI regions need to strengthen diagnostic networks and emergency care systems to mitigate global disparities in PUD burden.

In contrast to the established conclusion from prior research that males remain at persistently higher risk [22],our study reveals dynamic sex-specific patterns in the lifetime risks of PUD, with distinct inflection points in the sex ratio of risk observed across regions stratified by SDI. The earlier reversals in low-middle and low SDI regions may be attributable to socioeconomic disadvantages that disproportionately affect women, including malnutrition, particularly deficiencies in iron and other micronutrients that compromise gastric mucosal barrier function [52], multiparity-related stressors, and delays in screening and eradication of H. pylori infection [17, 18], leading to accelerated accumulation of lifetime risk of developing PUD early in life. In contrast, the delayed reversal in high-SDI regions likely reflects benefits from advanced socioeconomic development and medical technologies, which have substantially reduced overall lifetime risk of developing PUD; however, factors such as population aging, extended female life expectancy, increased NSAID use, and age-related declines in estrogen-mediated protection and gastric mucosal defenses ultimately culminate in female risk exceeding male risk at later time points [34, 55]. The protective role of estrogen is multifaceted. During reproductive years, estrogen contributes to gastric mucosal defense by inhibiting gastric acid secretion, upregulating cyclooxygenase-2 expression to promote the synthesis of cytoprotective prostaglandins, and enhancing mucosal blood flow and duodenal bicarbonate secretion to ensure adequate oxygen supply and neutralize retro-diffused hydrogen ions. The post-menopausal decline in estrogen thus removes this significant biological safeguard, unmasking women’s susceptibility to PUD in their later years [24, 56, 57]. Additionally, since 2020, the lifetime risk of dying from PUD has become higher for females than for males globally, with the exception of high-middle SDI regions. But in high-middle SDI regions, the sex ratio has been declining steadily and is expected to reverse around 2030.This finding challenges the longstanding assumption of persistently higher PUD mortality rates in males and underscores the need for heightened attention to women’s health in lower SDI regions. These findings underscore that global progress has been uneven, leaving low-SDI populations and women disproportionately vulnerable.

Furthermore, discrepancies exist between lifetime risk of developing PUD and the 0- to 74-year cumulative risk. Globally and across high-middle, middle, low-middle, and low-SDI regions, cumulative risk exceeded lifetime risk of developing, with the largest discrepancy observed in low-SDI regions, likely stemming from inadequate chronic disease management, lower survival rates among older adults, and shorter life expectancy. Conversely, in high-SDI regions, cumulative risk was lower than lifetime risk of developing, possibly reflecting better health care standards and longer life expectancy [8, 16].The use of cumulative risk assessment (ages 0–74 years) to estimate PUD burden may introduce bias due to variations in socioeconomic development, health care access, and life expectancy across regions. For instance, this method likely overestimates the actual burden in low-SDI regions while substantially underestimating it in high-SDI regions.

This study innovatively applies the lifetable method to quantify the lifetime risk of PUD, providing new indicators, revealing clear sex- and socioeconomic disparities and underscoring the need for stratified, population-specific policies.In low SDI regions, primary healthcare systems should be strengthened, and facilities should be equipped with rapid urease test reagents to conduct initial screening for H. pylori in patients with typical symptoms. Additionally, for women—particularly those of reproductive age, with multiparity, or experiencing undernutrition—gastrointestinal health assessment should be integrated into antenatal and perinatal services, with provision of selenium and zinc as well as iron supplementation and targeted dietary support. In high SDI regions, weight management policies need to be implemented, with anxiety screening incorporated into routine assessments in gastroenterology clinics. Meanwhile, regular remote endoscopic training should be provided to support low-resource regions. At the global level, PUD should be incorporated into WHO NCD and UHC indicator frameworks with sex- and poverty-disaggregated metrics; remote endoscopy training should be expanded; and interventions targeting women in low-SDI regions should be subject to ongoing evaluation.

While this study has limitations. First, reliance on GBD 2021 data, may introduce diagnostic bias in regions with underdeveloped health systems, potentially leading to overestimation or underestimation of risks in some areas. Second, the analysis reflects regional averages and may not fully capture subnational variations; differences in health care systems, diagnostic criteria, reporting practices, and public health policies could affect accuracy. Future research should validate these estimates using higher-quality epidemiological data and incorporate a broader range of socioeconomic and biological determinants, such as relevant biomarkers, to enhance precision.

## Conclusion

This study provides the first global assessment of lifetime risks of developing and dying from PUD, showing marked declines from 1990 to 2021. Yet, disparities remain across SDI regions and sexes. High-SDI regions face higher lifetime risk of developing from PUD but lower lifetime risk of dying, reflecting better healthcare access, while low-SDI regions show the opposite due to diagnostic and treatment gaps. Over time, the burden has shifted, with females surpassing males in several SDI regions. These results highlight the need for equitable, context-specific strategies, including wider H. pylori eradication, rational NSAIDs use, improved endoscopy, and targeted support for women in low-SDI regions. Integrating PUD care into universal health coverage is key to reducing burden and advancing health equity.

## Supplementary Information

Supplementary Material 1.

Supplementary Material 2.

Supplementary Material 3.

## Data Availability

The datasets generated and/or analyzed during the current study are available in the Global Burden of Disease, Injuries and Risk Factors Study.

## Abbreviations

PUD: Peptic ulcer disease
H. pylori: Helicobacter pylori
NSAIDs: Nonsteroidal anti-inflammatory drugs
PPI: Proton Pump Inhibitor
DALYs: Disability-adjusted life-years
SDI: Sociodemographic index
GBD: Global Burden of Disease
MR-BRT: Meta-regression with Bayesian priors, regularization, and trimming
ST-GPR: Spatiotemporal Gaussian process regression
ICD: International Classification of Diseases
GATHER: Guidelines for Accurate and Transparent Health Estimates Reporting
CI: Confidence interval
AAPC: Average annual percentage change

## References

[1] Almadi MA, Lu Y, Alali AA, Barkun AN. Peptic ulcer disease. Lancet. 2024;404(10447):68–81. 38885678 10.1016/S0140-6736(24)00155-7

[2] Lanas A, Chan FKL. Peptic ulcer disease. Lancet. 2017;390(10094):613–24. 28242110 10.1016/S0140-6736(16)32404-7

[3] Sverdén E, Agréus L, Dunn JM, Lagergren J. Peptic ulcer disease. BMJ. 2019;367:l5495. 31578179 10.1136/bmj.l5495

[4] Kavitt RT, Lipowska AM, Anyane-Yeboa A, Gralnek IM. Diagnosis and treatment of peptic ulcer disease. Am J Med. 2019;132(4):447–56. 30611829 10.1016/j.amjmed.2018.12.009

[5] Prabhu V, Shivani A. An overview of history, pathogenesis and treatment of perforated peptic ulcer disease with evaluation of prognostic scoring in adults. Ann Med Health Sci Res. 2014;4(1):22–9. 24669326 10.4103/2141-9248.126604 PMC3952291

[6] Gralnek IM, Dumonceau JM, Kuipers EJ, et al. Diagnosis and management of nonvariceal upper Gastrointestinal hemorrhage: European society of Gastrointestinal endoscopy (ESGE) guideline. Endoscopy. 2015;47(10):a1–46. 26417980 10.1055/s-0034-1393172

[7] Asaka M, Kato M, Sakamoto N. Roadmap to eliminate gastric cancer with *Helicobacter pylori* eradication and consecutive surveillance in Japan. J Gastroenterol. 2014;49(1):1–8. 24162382 10.1007/s00535-013-0897-8 PMC3895201

[8] GBD 2021 Diseases and Injuries Collaborators. Global incidence, prevalence, years lived with disability (YLDs), disability-adjusted life-years (DALYs), and healthy life expectancy (HALE) for 371 diseases and injuries in 204 countries and territories and 811 subnational locations, 1990–2021: a systematic analysis for the Global Burden of Disease Study 2021. Lancet. 2024;403(10440):2133–61. 38642570 10.1016/S0140-6736(24)00757-8 PMC11122111

[9] Everhart JE, Ruhl CE. Burden of digestive diseases in the united States part I: overall and upper gastrointestinal diseases. Gastroenterology. 2009;136(2):376–86. 19124023 10.1053/j.gastro.2008.12.015

[10] de Leest H, van Dieten H, van Tulder M, Lems WF, Dijkmans BAC, Boers M. Costs of treating bleeding and perforated peptic ulcers in the Netherlands. J Rheumatol. 2004;31(4):788–91. 15088309

[11] Dickerson JF, Salas SB, Donald J, et al. Economic burden of acute gastroenteritis among members of integrated healthcare delivery system, United States, 2014–2016. Emerg Infect Dis. 2024;30(5):968–73. 38666613 10.3201/eid3005.230356 PMC11060443

[12] Ahmad AS, Ormiston-Smith N, Sasieni PD. Trends in the lifetime risk of developing cancer in Great Britain: comparison of risk for those born from 1930 to 1960. Br J Cancer. 2015;112(5):943–7. 25647015 10.1038/bjc.2014.606 PMC4453943

[13] Sasieni PD, Shelton J, Ormiston-Smith N, Thomson CS, Silcocks PB. What is the lifetime risk of developing cancer? The effect of adjusting for multiple primaries. Br J Cancer. 2011;105(3):460–5. 21772332 10.1038/bjc.2011.250 PMC3172907

[14] NIH Consensus Conference. Helicobacter pylori in peptic ulcer disease. NIH Consensus Development Panel on Helicobacter pylori in Peptic Ulcer Disease. JAMA. 1994;272(1):65–69. 8007082

[15] Huang JQ, Sridhar S, Hunt RH. Role of *Helicobacter pylori* infection and non-steroidal anti-inflammatory drugs in peptic-ulcer disease: a meta-analysis. Lancet. 2002;359(9300):14–22. 11809181 10.1016/S0140-6736(02)07273-2

[16] Liu Q, Deng J, Yan W, et al. Burden and trends of infectious disease mortality attributed to air pollution, unsafe water, sanitation, and hygiene, and non-optimal temperature globally and in different socio-demographic index regions. Glob Health Res Policy. 2024;9(1):23. 38937833 10.1186/s41256-024-00366-x PMC11212388

[17] Archampong TN, Asmah RH, Richards CJ, et al. Gastro-duodenal disease in Africa: literature review and clinical data from Accra, Ghana. World J Gastroenterol. 2019;25(26):3344–58. 31341360 10.3748/wjg.v25.i26.3344 PMC6639557

[18] Smith S, Fowora M, Pellicano R. Infections with *Helicobacter pylori* and challenges encountered in Africa. World J Gastroenterol. 2019;25(25):3183–95. 31333310 10.3748/wjg.v25.i25.3183 PMC6626727

[19] Zhang Z, Yan W, Zhang X, et al. Peptic ulcer disease burden, trends, and inequalities in 204 countries and territories, 1990–2019: a population-based study. Therap Adv Gastroenterol. 2023;16:17562848231210375. 38026102 10.1177/17562848231210375 PMC10647969

[20] Ren J, Jin X, Li J, et al. The global burden of peptic ulcer disease in 204 countries and territories from 1990 to 2019: a systematic analysis for the Global Burden of Disease Study 2019. Int J Epidemiol. 2022;51(5):1666–76. 35234893 10.1093/ije/dyac033

[21] Xie X, Ren K, Zhou Z, Dang C, Zhang H. The global, regional and national burden of peptic ulcer disease from 1990 to 2019: a population-based study. BMC Gastroenterol. 2022;22(1):58. 35144540 10.1186/s12876-022-02130-2 PMC8832644

[22] Tan R, Zhao D, Zhang X, et al. Gender and age differences in the global burden of peptic ulcers: an analysis based on GBD data from 1990 to 2021. Front Med (Lausanne). 2025;12:1586270. 40357292 10.3389/fmed.2025.1586270 PMC12066501

[23] Vakil N. Peptic ulcer disease: a review. JAMA. 2024;332(21):1832–42. 39466269 10.1001/jama.2024.19094

[24] Chen KC, Kuo CY, Tsai WL, et al. Association between menopause, postmenopausal hormone therapy and peptic ulcer disease in Taiwanese population. Sci Rep. 2025;15(1):24199. Published 2025 Jul 7. 40624182 10.1038/s41598-025-08072-5 PMC12234781

[25] Chen W, Li Z, Zhao Y, Chen Y, Huang R. Global and national burden of atherosclerosis from 1990 to 2019: trend analysis based on the global burden of disease study 2019. Chin Med J. 2023;136(20):2442–50. 37677929 10.1097/CM9.0000000000002839 PMC10586830

[26] Bai Z, Wang H, Shen C, An J, Yang Z, Mo X. The global, regional, and national patterns of change in the burden of nonmalignant upper gastrointestinal diseases from 1990 to 2019 and the forecast for the next decade. Int J Surg. 2025;111(1):80–92. 38959095 10.1097/JS9.0000000000001902 PMC11745775

[27] Sung JJY, Kuipers EJ, El-Serag HB. Systematic review: the global incidence and prevalence of peptic ulcer disease. Aliment Pharmacol Ther. 2009;29(9):938–46. 19220208 10.1111/j.1365-2036.2009.03960.x

[28] Azhari H, King JA, Coward S, et al. The global incidence of peptic ulcer disease is decreasing since the turn of the 21st century: a study of the Organisation for Economic Co-Operation and Development (OECD). Am J Gastroenterol. 2022;117(9):1419–27. 35973143 10.14309/ajg.0000000000001843

[29] Nyssen OP, Bordin D, Tepes B, et al. European registry on Helicobacter pylori management (Hp-EuReg): patterns and trends in first-line empirical eradication prescription and outcomes of 5 years and 21 533 patients. Gut. 2021;70(1):40–54. 32958544 10.1136/gutjnl-2020-321372

[30] Chen YC, Malfertheiner P, Yu HT, et al. Global prevalence of Helicobacter pylori infection and incidence of gastric cancer between 1980 and 2022. Gastroenterology. 2024;166(4):605–19. 38176660 10.1053/j.gastro.2023.12.022

[31] Rocha GR, Lemos FFB, Silva LG, de O, et al. Overcoming antibiotic-resistant Helicobacter pylori infection: current challenges and emerging approaches. World J Gastroenterol. 2025;31(10):102289. 40093672 10.3748/wjg.v31.i10.102289 PMC11886534

[32] Abdul Rahim NR, Benson J, Grocke K et al. Prevalence of Helicobacter pylori infection in newly arrived refugees attending the migrant health Service, South Australia. Helicobacter. 2017;22(2). 10.1111/hel.12360. 10.1111/hel.12360 27717096

[33] Zhang W, Liang X, Chen X, Ge Z, Lu H. Time trends in the prevalence of *Helicobacter pylori* infection in patients with peptic ulcer disease: a single-center retrospective study in Shanghai. J Int Med Res. 2021;49(10):3000605211051167. 34686094 10.1177/03000605211051167 PMC8674481

[34] Szeto CC, Sugano K, Wang JG, et al. Non-steroidal anti-inflammatory drug (NSAID) therapy in patients with hypertension, cardiovascular, renal or gastrointestinal comorbidities: joint APAGE/APLAR/APSDE/APSH/APSN/PoA recommendations. Gut. 2020;69(4):617–29. 31937550 10.1136/gutjnl-2019-319300

[35] Schüssel K, Schulz M. Prescribing of COX-2 inhibitors in Germany after safety warnings and market withdrawals. Pharmazie. 2006;61(10):878–86. 17069430

[36] Rockwell MS, Oyese EG, Singh E, et al. Scoping review of interventions to de-implement potentially harmful non-steroidal anti-inflammatory drugs (NSAIDs) in healthcare settings. BMJ Open. 2024;14(4):e078808. 38631836 10.1136/bmjopen-2023-078808 PMC11029194

[37] Clarke K, Adler N, Agrawal D, et al. Indications for the use of proton pump inhibitors for stress ulcer prophylaxis and peptic ulcer bleeding in hospitalized patients. Am J Med. 2022;135(3):313–7. 34655535 10.1016/j.amjmed.2021.09.010

[38] Haastrup PF, Hansen JM, Søndergaard J, Jarbøl DE. Proton pump inhibitor use among patients at risk of peptic ulcer bleeding: a nationwide register-based study. Scand J Gastroenterol. 2021;56(1):6–12. 33280480 10.1080/00365521.2020.1853220

[39] Lin HJ. Role of proton pump inhibitors in the management of peptic ulcer bleeding. World J Gastrointest Pharmacol Ther. 2010;1(2):51–3. 21577296 10.4292/wjgpt.v1.i2.51 PMC3091149

[40] Gralnek IM, Stanley AJ, Morris AJ, et al. Endoscopic diagnosis and management of nonvariceal upper Gastrointestinal hemorrhage (NVUGIH): European society of Gastrointestinal endoscopy (ESGE) Guideline - Update 2021. Endoscopy. 2021;53(3):300–32. 33567467 10.1055/a-1369-5274

[41] Guo CLT, Wong SH, Lau LHS, et al. Timing of endoscopy for acute upper gastrointestinal bleeding: a territory-wide cohort study. Gut. 2022;71(8):1544–50. 34548338 10.1136/gutjnl-2020-323054 PMC9279843

[42] Cooper GS, Kou TD, Wong RCK. Use and impact of early endoscopy in elderly patients with peptic ulcer hemorrhage: a population-based analysis. Gastrointest Endosc. 2009;70(2):229–35. 19329112 10.1016/j.gie.2008.10.052

[43] Bygbjerg IC. Double burden of noncommunicable and infectious diseases in developing countries. Science. 2012;337(6101):1499–501. 22997329 10.1126/science.1223466

[44] Chen H, Song S, Cui R, Feng YW, Ge P. Global trends in *Staphylococcus aureus*-related lower respiratory infections from 1990 to 2021: findings from the 2021 global burden of disease report. Eur J Clin Microbiol Infect Dis. 2025;44(6):1455–69. 40186828 10.1007/s10096-025-05111-x

[45] Baker RE, Mahmud AS, Miller IF, et al. Infectious disease in an era of global change. Nat Rev Microbiol. 2022;20(4):193–205. 34646006 10.1038/s41579-021-00639-z PMC8513385

[46] Leone A, De la Fuente-Arrillaga C, Mas MV, et al. Association between the consumption of ultra-processed foods and the incidence of peptic ulcer disease in the SUN project: a Spanish prospective cohort study. Eur J Nutr. 2024;63(6):2367–78. 38809325 10.1007/s00394-024-03439-2 PMC11377682

[47] Melinder C, Udumyan R, Hiyoshi A, Brummer RJ, Montgomery S. Decreased stress resilience in young men significantly increases the risk of subsequent peptic ulcer disease-a prospective study of 233 093 men in Sweden. Aliment Pharmacol Ther. 2015;41(10):1005–15. 25809417 10.1111/apt.13168

[48] Bregonzio C, Armando I, Ando H, Jezova M, Baiardi G, Saavedra JM. Anti-inflammatory effects of angiotensin II AT1 receptor antagonism prevent stress-induced gastric injury. Am J Physiol Gastrointest Liver Physiol. 2003;285(2):G414–23. 12686508 10.1152/ajpgi.00058.2003

[49] Hamashima C, Systematic Review Group and Guideline Development Group for Gastric Cancer Screening Guidelines. Update version of the Japanese guidelines for gastric cancer screening. Jpn J Clin Oncol. 2018;48(7):673–83. 29889263 10.1093/jjco/hyy077

[50] Qin C, Liu Q, Wang Y, et al. Disease burden and geographic inequalities in 15 types of neonatal infectious diseases in 131 low- and middle-income countries and territories. Health Data Sci. 2024;4:0186. 39355853 10.34133/hds.0186 PMC11443844

[51] Liu Q, Jing W, Kang L, Liu J, Liu M. Trends of the global, regional and national incidence of malaria in 204 countries from 1990 to 2019 and implications for malaria prevention. J Travel Med. 2021;28(5):taab046. 33763689 10.1093/jtm/taab046 PMC8271200

[52] Hasan MM, Ahmed S, Soares Magalhaes RJ, Fatima Y, Biswas T, Mamun AA. Double burden of malnutrition among women of reproductive age in 55 low- and middle-income countries: progress achieved and opportunities for meeting the global target. Eur J Clin Nutr. 2022;76(2):277–87. 34040202 10.1038/s41430-021-00945-y PMC8152189

[53] Chao HC. Zinc deficiency and therapeutic value of zinc supplementation in pediatric gastrointestinal diseases. Nutrients. 2023;15(19):4093. 10.3390/nu15194093. 37836377 10.3390/nu15194093 PMC10574543

[54] Xu S, Kang Z, Li K, Li X, Zhang Y, Gao XJ. Selenium deficiency causes iron death and inflammatory injury through oxidative stress in the mice gastric mucosa. Biol Trace Elem Res. 2024;202(3):1150–63. 37394681 10.1007/s12011-023-03754-5

[55] GBD 2021 Rheumatoid Arthritis Collaborators. Global, regional, and National burden of rheumatoid arthritis, 1990–2020, and projections to 2050: a systematic analysis of the global burden of disease study 2021. Lancet Rheumatol. 2023;5(10):e594–610. 37795020 10.1016/S2665-9913(23)00211-4 PMC10546867

[56] Shore R, Björne H, Omoto Y, et al. Sex differences and effects of oestrogen in rat gastric mucosal defence. World J Gastroenterol. 2017;23(3):426–36. 28210078 10.3748/wjg.v23.i3.426 PMC5291847

[57] Nie X, Xie R, Tuo B. Effects of estrogen on the gastrointestinal tract. Dig Dis Sci. 2018;63(3):583–96. 29387989 10.1007/s10620-018-4939-1

