# Supplementary material for: Socioeconomic inequalities and dynamic changes in sex differences in lifetime risks of peptic ulcer disease
Source: Biol Sex Differ. 2026 Jan 27;17:33. doi: 10.1186/s13293-026-00832-w (PMC12918435; doi:10.1186/s13293-026-00832-w)
Supplement: Supplementary file 1 — Supplementary Material 1. [file 13293_2026_832_MOESM1_ESM.docx]

**1.Lifetime risk of developing and dying from PUD
1.1 Formulas**

$$p_{lifetime risk of developing}=\frac{1}{\mathcal{l}_{0}} \sum_{x=1}^{w} L_{x}.I_{x}$$

$$p_{lifetime risk of dying}=\frac{1}{\mathcal{l}_{0}} \sum_{x=1}^{w} L_{x}.D_{x}$$

$\mathcal{l}_{0}$ represents the initial population size at the beginning of the first age interval in the lifetable.
𝑥 represents the age group, here defined as a 5-year interval.
$w$ represents the oldest age group considered in the study.
I_x_ is the incidence of peptic ulcer disease in age group 𝑥,indicating the number of new cases of peptic ulcer disease in that age group.
D_x_ is the mortality rate in age group 𝑥,representing the probability of death from PUD in that age group.
L_x_ is the life expectancy at age group 𝑥,derived from the general population lifetable.

**1.2 Adjustment for Competing Risks:**

This method inherently accounts for competing risks through the use of the key parameter L_x_. The parameter L_x_, which incorporates information on all-cause mortality, decreases progressively as cohort members are lost to death from all causes, including competing risks. Thus, during the summation process in the formula, the influence of competing risks is automatically and fully accounted for. The lifetime risk we report represents a realistic estimate of an individual’s actual risk under real-world conditions where competing risks are present. **Reference:**[1] Ahmad AS, Ormiston-Smith N, Sasieni PD. Trends in the lifetime risk of developing cancer in Great Britain: comparison of risk for those born from 1930 to 1960. Br J Cancer. 2015;112(5):943-947.

[2] Sasieni PD, Shelton J, Ormiston-Smith N, Thomson CS, Silcocks PB. What is the lifetime risk of developing cancer?: the effect of adjusting for multiple primaries. Br J Cancer. 2011;105(3):460-465.
[3]Esteve J, Benhamou E, Raymond L (1994) Descriptive Epidemiology. International Agency for Research on Cancer. (IARC Scientific Publications No. 128): Lyon, pp 67–68

**2.** **Average Annual Percent Change (AAPC)
2.1 Formulas**The AAPC over any fixed interval is calculated using a weighted average of the slope coefficients of the underlying joinpoint regression model with the weights equal to the length of each segment over the interval. The final step of the calculation transforms the weighted average of slope coefficients to an annual percent change. If we denote bi as the slope coefficient for the I th segment with i indexing the segments in the desired range of years, and wi as the length of each segment in the range of years, then:

APC_i_ = { exp(bi) - 1 } x 100
AAPC = { exp($\frac{\sum{w_{i}b}_{i}}{{\sum w}_{i}}$) - 1 } x 100
bi: as the slope coefficient for the I th segment with i indexing the segments in the desired range of years
wi: as the length of each segment in the range of years

Reference:
<https://surveillance.cancer.gov/help/joinpoint/setting-parameters/method-and-parameters-tab/apc-aapc-tau-confidence-intervals/average-annual-percent-change-aapc>
