# Supplementary material for: Socioeconomic inequalities and dynamic changes in sex differences in lifetime risks of peptic ulcer disease
Source: Biol Sex Differ. 2026 Jan 27;17:33. doi: 10.1186/s13293-026-00832-w (PMC12918435; doi:10.1186/s13293-026-00832-w)
Supplement: Supplementary file 3 — Supplementary Material 3. [file 13293_2026_832_MOESM3_ESM.docx]

|  | **TableS1**.Lifetime risks(%) of developing and dying from PUD in 1990 and 2021,male | | | | | |  |
| --- | --- | --- | --- | --- | --- | --- | --- |
| Location | | LRDE(95% CI) | | AAPC,1990-2021 | LRDY(95% CI) | | AAPC,1990-2021 |
|  |  | 1990 | 2021 |  | 1990 | 2021 |  |
| Global | | 4.99(4.98-5.01) | 3.21(3.20,3.22) | -1.43 (-1.53, -1.33) | 0.79(0.78,0.79) | 0.34(0.34,0.34) | -2.73 (-2.86, -2.60) |
| High SDI | | 4.87(4.84-4.91) | 3.77(3.73,3.82) | -0.82 (-0.91, -0.74) | 0.53(0.52,0.53) | 0.22(0.22,0.23) | -2.84 (-3.32, -2.35) |
| High-middle SDI | | 4.74(4.71-4.77) | 3.22(3.19,3.25) | -1.23 (-1.37, -1.09) | 0.58(0.57,0.59) | 0.31(0.30,0.31) | -2.11 (-2.42, -1.80) |
| Middle SDI | | 5.55(5.52-5.57) | 3.27(3.25,3.28) | -1.71 (-1.83, -1.60) | 0.82(0.81,0.83) | 0.33(0.33,0.34) | -2.90 (-3.03, -2.77) |
| Low-middle SDI | | 5.56(5.53-5.59) | 2.93(2.91,2.95) | -2.06 (-2.19, -1.93) | 1.25(1.24,1.26) | 0.44(0.44,0.44) | -3.31 (-3.52, -3.10) |
| Low SDI | | 3.82(3.79-3.85) | 2.66(2.64,2.68) | -1.13 (-1.47, -0.79) | 1.04(1.02,1.05) | 0.47(0.46,0.48) | -2.66 (-3.08, -2.24) |
| Regional | |  |  |  |  |  |  |
| Southern Latin America | | 2.11(2.04-2.17) | 1.61(1.55,1.66) | -0.91 (-1.07, -0.75) | 0.45(0.42,0.48) | 0.18(0.16,0.20) | -2.88 (-3.87, -1.88) |
| Western Europe | | 2.98(2.95-3.01) | 1.95(1.90,1.99) | -1.37 (-1.43, -1.30) | 0.57(0.56,0.59) | 0.21(0.21,0.22) | -3.25 (-3.76, -2.74) |
| High-income North America | | 5.40(5.35-5.46) | 4.22(4.17,4.28) | -0.82 (-0.95, -0.68) | 0.32(0.31,0.33) | 0.12(0.11,0.12) | -3.20 (-3.69, -2.72) |
| Australasia | | 3.78(3.62-3.93) | 1.99(1.85,2.13) | -2.07 (-2.21, -1.93) | 0.71(0.65,0.78) | 0.19(0.16,0.21) | -4.16 (-4.88, -3.44) |
| High-income Asia Pacific | | 6.60(6.45-6.75) | 5.42(5.10,5.74) | -0.65 (-0.87, -0.43) | 0.61(0.59,0.64) | 0.24(0.23,0.25) | -2.91 (-3.19, -2.64) |
| Caribbean | | 3.42(3.28-3.55) | 2.30(2.20,2.40) | -1.17 (-1.42, -0.92) | 0.92(0.86,0.98) | 0.39(0.36,0.41) | -2.88 (-3.41, -2.34) |
| Central Latin America | | 3.33(3.26-3.39) | 1.61(1.59,1.64) | -2.49 (-2.68, -2.30) | 1.11(1.07,1.15) | 0.32(0.31,0.33) | -4.03 (-4.48, -3.57) |
| Tropical Latin America | | 4.02(3.96-4.08) | 1.91(1.88,1.94) | -2.37 (-2.59, -2.15) | 0.60(0.57,0.62) | 0.30(0.29,0.31) | -2.21 (-2.48, -1.95) |
| Andean Latin America | | 3.70(3.55-3.84) | 1.79(1.73,1.86) | -2.46 (-2.69, -2.23) | 1.12(1.04,1.20) | 0.20(0.18,0.21) | -5.72 (-6.39, -5.05) |
| Central Sub-Saharan Africa | | 2.49(2.42-2.57) | 2.17(2.12,2.22) | -0.45 (-0.58, -0.32) | 0.62(0.58,0.65) | 0.43(0.40,0.45) | -1.26 (-1.48, -1.03) |
| Eastern Sub-Saharan Africa | | 2.63(2.59-2.66) | 2.34(2.31,2.37) | 0.12 (-0.06, 0.31) | 0.88(0.86,0.90) | 0.53(0.51,0.54) | -1.43 (-2.46, -0.38) |
| Southern Sub-Saharan Africa | | 2.93(2.84-3.02) | 1.93(1.89,1.97) | -1.34 (-1.70, -0.98) | 0.55(0.50,0.59) | 0.24(0.23,0.25) | -2.66 (-2.92, -2.39) |
| Western Sub-Saharan Africa | | 2.88(2.83-2.93) | 2.67(2.63,2.70) | -0.29 (-0.39, -0.19) | 0.57(0.55,0.59) | 0.33(0.32,0.34) | -1.77 (-2.07, -1.47) |
| North Africa and Middle East | | 3.03(2.99-3.07) | 2.16(2.14,2.19) | -1.11 (-1.18, -1.03) | 0.63(0.61,0.65) | 0.20(0.20,0.21) | -3.70 (-3.90, -3.50) |
| South Asia | | 6.32(6.29-6.36) | 2.89(2.88,2.91) | -2.50 (-2.66, -2.35) | 1.39(1.38,1.40) | 0.44(0.43,0.44) | -3.69 (-3.93, -3.46) |
| East Asia | | 6.90(6.86-6.93) | 4.35(4.31,4.38) | -1.50 (-1.65, -1.34) | 0.86(0.85,0.87) | 0.38(0.37,0.38) | -2.66 (-2.95, -2.37) |
| Southeast Asia | | 4.47(4.43-4.52) | 3.24(3.21,3.28) | -1.02 (-1.15, -0.88) | 0.73(0.71,0.74) | 0.37(0.36,0.37) | -2.13 (-2.31, -1.96) |
| Oceania(Expect Australasia) | | 6.08(5.59-6.57) | 4.60(4.28,4.93) | -0.85 (-1.02, -0.67) | 0.96(0.80,1.11) | 0.49(0.43,0.56) | -2.05 (-2.25, -1.84) |
| Central Asia | | 3.42(3.33-3.51) | 3.01(2.92,3.10) | -2.50 (-2.66, -2.35) | 0.55(0.52,0.58) | 0.40(0.38,0.42) | -1.20 (-1.72, -0.67) |
| Eastern Europe | | 4.19(4.14-4.23) | 3.69(3.64,3.75) | -0.46 (-0.87, -0.05) | 0.49(0.48,0.50) | 0.47(0.46,0.48) | -0.07 (-0.78, 0.66) |
| Central Europe | | 4.17(4.10-4.23) | 3.62(3.51,3.74) | -0.43 (-0.63, -0.23) | 0.61(0.59,0.62) | 0.46(0.44,0.47) | -0.92 (-1.28, -0.55) |

LRDE=lifetime risk of developing(%); LRDY=lifetime risk of dying(%);CI = confidence interval;SDI:sociodemographic index

**Table S2**.Lifetime risks(%) of developing and dying from PUD in 1990 and 2021,female

| Location | LRDE(95% CI) | | AAPC,1990-2021 | LRDY(95% CI) | | AAPC,1990-2021 |
| --- | --- | --- | --- | --- | --- | --- |
|  | 1990 | 2021 |  | 1990 | 2021 |  |
| Global | 4.37(4.36-4.39) | 3.21(3.20,3.23) | -1.00 (-1.10, -0.89) | 0.61(0.60,0.61) | 0.35(0.35,0.36) | -1.80 (-2.00, -1.60) |
| High SDI | 4.63(4.58-4.68) | 3.92(3.85,3.98) | -0.54 (-0.66, -0.42) | 0.53(0.52,0.53) | 0.23(0.23,0.23) | -2.69 (-2.91, -2.48) |
| High-middle SDI | 3.33(3.30-3.36) | 2.55(2.51,2.59) | -0.83 (-0.92, -0.73) | 0.34(0.33,0.34) | 0.26(0.26,0.27) | -0.85 (-1.36, -0.35) |
| Middle SDI | 4.84(4.81-4.86) | 3.02(3.00,3.04) | -1.51 (-1.68, -1.33) | 0.70(0.69,0.71) | 0.37(0.36,0.37) | -2.06 (-2.35, -1.77) |
| Low-middle SDI | 5.33(5.30-5.36) | 3.52(3.49,3.54) | -1.35 (-1.54, -1.16) | 0.97(0.96,0.98) | 0.51(0.51,0.52) | -2.05 (-2.25, -1.84) |
| Low SDI | 3.49(3.46-3.53) | 2.89(2.87,2.92) | -0.63 (-0.74, -0.52) | 0.86(0.85,0.88) | 0.55(0.54,0.56) | -1.48 (-1.89, -1.06) |
| Regional |  |  |  |  |  |  |
| Southern Latin America | 1.61(1.55-1.68) | 1.36(1.30,1.42) | -0.54 (-0.67, -0.41) | 0.29(0.27,0.32) | 0.16(0.14,0.17) | -1.84 (-2.91, -0.76) |
| Western Europe | 2.64(2.61-2.68) | 1.77(1.72,1.83) | -1.32 (-1.48, -1.16) | 0.56(0.55,0.57) | 0.21(0.20,0.21) | -3.17 (-3.36, -2.98) |
| High-income North America | 6.28(6.18-6.38) | 5.30(5.20,5.40) | -0.59 (-0.77, -0.40) | 0.35(0.34,0.36) | 0.13(0.12,0.13) | -3.20 (-3.96, -2.45) |
| Australasia | 4.13(3.93-4.33) | 2.01(1.81,2.20) | -2.31 (-2.43, -2.19) | 0.86(0.79,0.94) | 0.20(0.17,0.23) | -4.50 (-4.83, -4.17) |
| High-income Asia Pacific | 5.30(5.17-5.43) | 4.07(3.80,4.33) | -0.84 (-1.01, -0.66) | 0.67(0.65,0.70) | 0.24(0.23,0.25) | -3.29 (-3.76, -2.81) |
| Caribbean | 2.44(2.27-2.61) | 1.91(1.75,2.06) | -0.65 (-1.32, 0.02) | 0.54(0.49,0.59) | 0.27(0.25,0.30) | -1.86 (-2.24, -1.47) |
| Central Latin America | 3.20(3.13-3.27) | 1.73(1.71,1.76) | -1.97 (-2.14, -1.81) | 1.22(1.17,1.27) | 0.38(0.36,0.39) | -3.77 (-4.15, -3.39) |
| Tropical Latin America | 3.36(3.28-3.43) | 1.71(1.68,1.75) | -2.13 (-2.47, -1.78) | 0.44(0.41,0.46) | 0.26(0.25,0.27) | -1.67 (-2.13, -1.22) |
| Andean Latin America | 3.49(3.31-3.67) | 2.03(1.95,2.11) | -1.68 (-2.06, -1.31) | 0.95(0.87,1.03) | 0.24(0.22,0.26) | -4.50 (-5.11, -3.90) |
| Central Sub-Saharan Africa | 2.19(2.10-2.27) | 2.46(2.39,2.54) | 0.36 (0.31, 0.40) | 0.45(0.41,0.49) | 0.42(0.40,0.44) | -0.25 (-0.46, -0.04) |
| Eastern Sub-Saharan Africa | 1.64(1.60-1.67) | 1.59(1.56,1.62) | -0.13 (-0.32, 0.07) | 0.55(0.53,0.57) | 0.44(0.42,0.45) | -0.66 (-1.18, -0.13) |
| Southern Sub-Saharan Africa | 2.46(2.37-2.56) | 1.77(1.72,1.82) | -1.08 (-1.49, -0.67) | 0.60(0.54,0.65) | 0.29(0.28,0.31) | -2.37 (-2.72, -2.01) |
| Western Sub-Saharan Africa | 2.93(2.86-2.99) | 3.31(3.26,3.37) | 0.40 (0.33, 0.47) | 0.57(0.55,0.59) | 0.56(0.54,0.58) | -0.10 (-0.32, 0.12) |
| North Africa and Middle East | 3.14(3.09-3.19) | 2.72(2.67,2.77) | -0.49 (-0.64, -0.35) | 0.47(0.45,0.48) | 0.22(0.21,0.23) | -2.50 (-2.77, -2.23) |
| South Asia | 6.36(6.32-6.39) | 3.78(3.76,3.81) | -1.66 (-1.88, -1.45) | 1.21(1.20,1.23) | 0.59(0.59,0.60) | -2.32 (-2.50, -2.14) |
| East Asia | 5.64(5.61-5.68) | 3.40(3.36,3.44) | -1.64 (-1.71, -1.56) | 0.62(0.61,0.62) | 0.31(0.31,0.32) | -2.23 (-2.55, -1.91) |
| Southeast Asia | 4.25(4.21-4.30) | 3.08(3.04,3.13) | -0.95 (-1.36, -0.54) | 0.67(0.65,0.69) | 0.42(0.41,0.43) | -1.44 (-1.64, -1.23) |
| Oceania(Expect Australasia) | 5.15(4.60-5.71) | 4.59(4.17,5.01) | -0.33 (-0.45, -0.21) | 0.67(0.51,0.83) | 0.38(0.31,0.46) | -1.74 (-2.00, -1.47) |
| Central Asia | 2.18(2.09-2.28) | 2.48(2.37,2.59) | 0.40 (0.28, 0.52) | 0.23(0.21,0.25) | 0.28(0.26,0.30) | 0.58 (-0.09, 1.25) |
| Eastern Europe | 2.00(1.96-2.04) | 2.47(2.41,2.54) | 0.70 (0.38, 1.02) | 0.19(0.18,0.20) | 0.36(0.36,0.37) | 2.08 (1.52, 2.64) |
| Central Europe | 3.59(3.47-3.70) | 3.55(3.34,3.76) | -0.01 (-0.11, 0.09) | 0.40(0.38,0.42) | 0.42(0.40,0.43) | 0.14 (-0.35, 0.63) |

LRDE=lifetime risk of developing(%); LRDY=lifetime risk of dying(%);CI = confidence interval;SDI:sociodemographic index

| **Table S3**.The gender ratio of global and regional lifetime risks of developing PUD from 1990 to 2021 | | |
| --- | --- | --- |
| year | location_name | gender_ratio |
| 1990 | Global | 1.14(1.14-1.15) |
| 1991 | Global | 1.15(1.14-1.15) |
| 1992 | Global | 1.15(1.14-1.15) |
| 1993 | Global | 1.14(1.14-1.15) |
| 1994 | Global | 1.14(1.13-1.14) |
| 1995 | Global | 1.14(1.13-1.15) |
| 1996 | Global | 1.13(1.13-1.14) |
| 1997 | Global | 1.13(1.12-1.13) |
| 1998 | Global | 1.11(1.11-1.12) |
| 1999 | Global | 1.10(1.10-1.11) |
| 2000 | Global | 1.09(1.09-1.09) |
| 2001 | Global | 1.09(1.08-1.09) |
| 2002 | Global | 1.08(1.08-1.09) |
| 2003 | Global | 1.08(1.07-1.09) |
| 2004 | Global | 1.08(1.07-1.08) |
| 2005 | Global | 1.07(1.07-1.08) |
| 2006 | Global | 1.07(1.07-1.08) |
| 2007 | Global | 1.07(1.06-1.07) |
| 2008 | Global | 1.06(1.06-1.07) |
| 2009 | Global | 1.06(1.06-1.07) |
| 2010 | Global | 1.06(1.05-1.06) |
| 2011 | Global | 1.05(1.05-1.06) |
| 2012 | Global | 1.05(1.04-1.05) |
| 2013 | Global | 1.05(1.04-1.05) |
| 2014 | Global | 1.04(1.04-1.05) |
| 2015 | Global | 1.04(1.03-1.05) |
| 2016 | Global | 1.04(1.03-1.04) |
| 2017 | Global | 1.03(1.03-1.04) |
| 2018 | Global | 1.03(1.02-1.03) |
| 2019 | Global | 1.02(1.02-1.03) |
| 2020 | Global | 1.01(1.00-1.01) |
| 2021 | Global | 1.00(0.99-1.00) |
| 1990 | High SDI | 1.05(1.04-1.07) |
| 1991 | High SDI | 1.06(1.04-1.07) |
| 1992 | High SDI | 1.06(1.05-1.08) |
| 1993 | High SDI | 1.06(1.05-1.08) |
| 1994 | High SDI | 1.06(1.05-1.08) |
| 1995 | High SDI | 1.06(1.05-1.08) |
| 1996 | High SDI | 1.06(1.04-1.07) |
| 1997 | High SDI | 1.05(1.03-1.06) |
| 1998 | High SDI | 1.03(1.02-1.05) |
| 1999 | High SDI | 1.02(1.00-1.03) |
| 2000 | High SDI | 1.01(1.00-1.03) |
| 2001 | High SDI | 1.01(1.00-1.03) |
| 2002 | High SDI | 1.02(1.01-1.04) |
| 2003 | High SDI | 1.04(1.02-1.05) |
| 2004 | High SDI | 1.04(1.03-1.06) |
| 2005 | High SDI | 1.04(1.03-1.06) |
| 2006 | High SDI | 1.04(1.02-1.06) |
| 2007 | High SDI | 1.04(1.02-1.06) |
| 2008 | High SDI | 1.03(1.02-1.05) |
| 2009 | High SDI | 1.03(1.01-1.05) |
| 2010 | High SDI | 1.03(1.01-1.05) |
| 2011 | High SDI | 1.03(1.01-1.05) |
| 2012 | High SDI | 1.03(1.01-1.05) |
| 2013 | High SDI | 1.02(1.00-1.05) |
| 2014 | High SDI | 1.02(1.00-1.04) |
| 2015 | High SDI | 1.01(0.99-1.04) |
| 2016 | High SDI | 1.01(0.99-1.03) |
| 2017 | High SDI | 1.00(0.97-1.02) |
| 2018 | High SDI | 0.98(0.96-1.01) |
| 2019 | High SDI | 0.98(0.95-1.00) |
| 2020 | High SDI | 0.98(0.96-1.00) |
| 2021 | High SDI | 0.96(0.94-0.99) |
| 1990 | High-middle SDI | 1.42(1.41-1.44) |
| 1991 | High-middle SDI | 1.42(1.41-1.44) |
| 1992 | High-middle SDI | 1.42(1.40-1.43) |
| 1993 | High-middle SDI | 1.41(1.39-1.43) |
| 1994 | High-middle SDI | 1.41(1.39-1.42) |
| 1995 | High-middle SDI | 1.40(1.39-1.42) |
| 1996 | High-middle SDI | 1.40(1.39-1.42) |
| 1997 | High-middle SDI | 1.41(1.39-1.42) |
| 1998 | High-middle SDI | 1.40(1.38-1.42) |
| 1999 | High-middle SDI | 1.39(1.37-1.40) |
| 2000 | High-middle SDI | 1.37(1.35-1.39) |
| 2001 | High-middle SDI | 1.36(1.34-1.37) |
| 2002 | High-middle SDI | 1.35(1.33-1.36) |
| 2003 | High-middle SDI | 1.33(1.32-1.35) |
| 2004 | High-middle SDI | 1.31(1.30-1.33) |
| 2005 | High-middle SDI | 1.30(1.29-1.32) |
| 2006 | High-middle SDI | 1.31(1.29-1.32) |
| 2007 | High-middle SDI | 1.31(1.29-1.32) |
| 2008 | High-middle SDI | 1.31(1.29-1.33) |
| 2009 | High-middle SDI | 1.31(1.29-1.33) |
| 2010 | High-middle SDI | 1.31(1.29-1.33) |
| 2011 | High-middle SDI | 1.31(1.29-1.33) |
| 2012 | High-middle SDI | 1.30(1.28-1.32) |
| 2013 | High-middle SDI | 1.30(1.28-1.32) |
| 2014 | High-middle SDI | 1.29(1.27-1.31) |
| 2015 | High-middle SDI | 1.29(1.27-1.31) |
| 2016 | High-middle SDI | 1.28(1.26-1.30) |
| 2017 | High-middle SDI | 1.28(1.26-1.30) |
| 2018 | High-middle SDI | 1.28(1.25-1.30) |
| 2019 | High-middle SDI | 1.27(1.25-1.29) |
| 2020 | High-middle SDI | 1.26(1.23-1.29) |
| 2021 | High-middle SDI | 1.26(1.24-1.29) |
| 1990 | Middle SDI | 1.15(1.14-1.16) |
| 1991 | Middle SDI | 1.15(1.14-1.16) |
| 1992 | Middle SDI | 1.16(1.15-1.17) |
| 1993 | Middle SDI | 1.16(1.15-1.17) |
| 1994 | Middle SDI | 1.16(1.15-1.17) |
| 1995 | Middle SDI | 1.16(1.15-1.17) |
| 1996 | Middle SDI | 1.15(1.14-1.16) |
| 1997 | Middle SDI | 1.14(1.13-1.15) |
| 1998 | Middle SDI | 1.13(1.12-1.14) |
| 1999 | Middle SDI | 1.13(1.12-1.14) |
| 2000 | Middle SDI | 1.12(1.11-1.13) |
| 2001 | Middle SDI | 1.12(1.11-1.13) |
| 2002 | Middle SDI | 1.12(1.11-1.12) |
| 2003 | Middle SDI | 1.11(1.10-1.12) |
| 2004 | Middle SDI | 1.11(1.10-1.12) |
| 2005 | Middle SDI | 1.11(1.10-1.12) |
| 2006 | Middle SDI | 1.11(1.10-1.12) |
| 2007 | Middle SDI | 1.11(1.10-1.12) |
| 2008 | Middle SDI | 1.12(1.11-1.12) |
| 2009 | Middle SDI | 1.12(1.11-1.13) |
| 2010 | Middle SDI | 1.12(1.11-1.13) |
| 2011 | Middle SDI | 1.12(1.11-1.13) |
| 2012 | Middle SDI | 1.11(1.10-1.12) |
| 2013 | Middle SDI | 1.11(1.10-1.12) |
| 2014 | Middle SDI | 1.11(1.10-1.12) |
| 2015 | Middle SDI | 1.11(1.10-1.12) |
| 2016 | Middle SDI | 1.11(1.10-1.12) |
| 2017 | Middle SDI | 1.10(1.09-1.11) |
| 2018 | Middle SDI | 1.10(1.09-1.11) |
| 2019 | Middle SDI | 1.10(1.09-1.11) |
| 2020 | Middle SDI | 1.08(1.07-1.09) |
| 2021 | Middle SDI | 1.08(1.07-1.09) |
| 1990 | Low-middle SDI | 1.04(1.03-1.05) |
| 1991 | Low-middle SDI | 1.04(1.03-1.05) |
| 1992 | Low-middle SDI | 1.04(1.03-1.05) |
| 1993 | Low-middle SDI | 1.03(1.02-1.04) |
| 1994 | Low-middle SDI | 1.03(1.02-1.04) |
| 1995 | Low-middle SDI | 1.03(1.02-1.03) |
| 1996 | Low-middle SDI | 1.02(1.01-1.02) |
| 1997 | Low-middle SDI | 1.00(0.99-1.01) |
| 1998 | Low-middle SDI | 0.99(0.98-1.00) |
| 1999 | Low-middle SDI | 0.98(0.97-0.98) |
| 2000 | Low-middle SDI | 0.96(0.95-0.97) |
| 2001 | Low-middle SDI | 0.95(0.94-0.96) |
| 2002 | Low-middle SDI | 0.94(0.93-0.95) |
| 2003 | Low-middle SDI | 0.93(0.92-0.94) |
| 2004 | Low-middle SDI | 0.92(0.92-0.93) |
| 2005 | Low-middle SDI | 0.92(0.92-0.93) |
| 2006 | Low-middle SDI | 0.92(0.91-0.93) |
| 2007 | Low-middle SDI | 0.91(0.90-0.92) |
| 2008 | Low-middle SDI | 0.91(0.90-0.91) |
| 2009 | Low-middle SDI | 0.90(0.89-0.91) |
| 2010 | Low-middle SDI | 0.89(0.88-0.90) |
| 2011 | Low-middle SDI | 0.89(0.88-0.89) |
| 2012 | Low-middle SDI | 0.89(0.88-0.89) |
| 2013 | Low-middle SDI | 0.89(0.88-0.89) |
| 2014 | Low-middle SDI | 0.88(0.87-0.89) |
| 2015 | Low-middle SDI | 0.88(0.87-0.89) |
| 2016 | Low-middle SDI | 0.88(0.87-0.89) |
| 2017 | Low-middle SDI | 0.87(0.87-0.88) |
| 2018 | Low-middle SDI | 0.87(0.86-0.88) |
| 2019 | Low-middle SDI | 0.87(0.86-0.88) |
| 2020 | Low-middle SDI | 0.84(0.83-0.85) |
| 2021 | Low-middle SDI | 0.83(0.82-0.84) |
| 1990 | Low SDI | 1.09(1.08-1.11) |
| 1991 | Low SDI | 1.10(1.09-1.12) |
| 1992 | Low SDI | 1.11(1.10-1.13) |
| 1993 | Low SDI | 1.11(1.10-1.13) |
| 1994 | Low SDI | 1.05(1.04-1.06) |
| 1995 | Low SDI | 1.11(1.10-1.12) |
| 1996 | Low SDI | 1.10(1.09-1.11) |
| 1997 | Low SDI | 1.09(1.07-1.10) |
| 1998 | Low SDI | 1.07(1.06-1.09) |
| 1999 | Low SDI | 1.06(1.05-1.08) |
| 2000 | Low SDI | 1.06(1.04-1.07) |
| 2001 | Low SDI | 1.05(1.04-1.07) |
| 2002 | Low SDI | 1.05(1.04-1.07) |
| 2003 | Low SDI | 1.05(1.03-1.06) |
| 2004 | Low SDI | 1.04(1.02-1.05) |
| 2005 | Low SDI | 1.03(1.02-1.04) |
| 2006 | Low SDI | 1.03(1.01-1.04) |
| 2007 | Low SDI | 1.02(1.00-1.03) |
| 2008 | Low SDI | 1.01(1.00-1.02) |
| 2009 | Low SDI | 1.00(0.99-1.02) |
| 2010 | Low SDI | 1.00(0.99-1.01) |
| 2011 | Low SDI | 0.99(0.98-1.01) |
| 2012 | Low SDI | 1.00(0.98-1.01) |
| 2013 | Low SDI | 0.99(0.98-1.00) |
| 2014 | Low SDI | 0.99(0.97-1.00) |
| 2015 | Low SDI | 0.98(0.97-0.99) |
| 2016 | Low SDI | 0.97(0.96-0.98) |
| 2017 | Low SDI | 0.96(0.95-0.98) |
| 2018 | Low SDI | 0.96(0.95-0.97) |
| 2019 | Low SDI | 0.96(0.94-0.97) |
| 2020 | Low SDI | 0.93(0.91-0.94) |
| 2021 | Low SDI | 0.92(0.91-0.93) |

| **TableS4**.The gender ratio of global and regional lifetime risks of dying PUD from1990 to 2021 | | |
| --- | --- | --- |
| year | location_name | gender ratio |
| 1990 | Global | 1.30(1.28-1.31) |
| 1991 | Global | 1.28(1.27-1.30) |
| 1992 | Global | 1.28(1.27-1.30) |
| 1993 | Global | 1.27(1.25-1.28) |
| 1994 | Global | 1.25(1.24-1.26) |
| 1995 | Global | 1.25(1.24-1.26) |
| 1996 | Global | 1.25(1.23-1.26) |
| 1997 | Global | 1.23(1.22-1.25) |
| 1998 | Global | 1.23(1.22-1.24) |
| 1999 | Global | 1.22(1.21-1.24) |
| 2000 | Global | 1.21(1.19-1.23) |
| 2001 | Global | 1.19(1.17-1.21) |
| 2002 | Global | 1.16(1.15-1.17) |
| 2003 | Global | 1.14(1.14-1.15) |
| 2004 | Global | 1.14(1.12-1.15) |
| 2005 | Global | 1.12(1.12-1.12) |
| 2006 | Global | 1.09(1.08-1.10) |
| 2007 | Global | 1.08(1.08-1.08) |
| 2008 | Global | 1.07(1.06-1.08) |
| 2009 | Global | 1.08(1.07-1.10) |
| 2010 | Global | 1.08(1.06-1.09) |
| 2011 | Global | 1.08(1.07-1.09) |
| 2012 | Global | 1.04(1.03-1.06) |
| 2013 | Global | 1.04(1.03-1.06) |
| 2014 | Global | 1.04(1.03-1.06) |
| 2015 | Global | 1.03(1.02-1.04) |
| 2016 | Global | 1.02(1.01-1.04) |
| 2017 | Global | 1.02(1.02-1.02) |
| 2018 | Global | 1.01(1.00-1.02) |
| 2019 | Global | 1.01(1.00-1.02) |
| 2020 | Global | 0.97(0.97-0.97) |
| 2021 | Global | 0.96(0.95-0.97) |
| 1990 | High SDI | 1.00(0.99-1.01) |
| 1991 | High SDI | 0.99(0.97-1.01) |
| 1992 | High SDI | 0.97(0.95-0.99) |
| 1993 | High SDI | 0.95(0.93-0.97) |
| 1994 | High SDI | 0.96(0.93-0.99) |
| 1995 | High SDI | 0.96(0.94-0.97) |
| 1996 | High SDI | 0.95(0.92-0.97) |
| 1997 | High SDI | 0.94(0.92-0.97) |
| 1998 | High SDI | 0.97(0.94-0.99) |
| 1999 | High SDI | 0.98(0.96-0.99) |
| 2000 | High SDI | 0.95(0.93-0.97) |
| 2001 | High SDI | 0.94(0.91-0.97) |
| 2002 | High SDI | 0.95(0.91-0.99) |
| 2003 | High SDI | 0.95(0.93-0.97) |
| 2004 | High SDI | 0.92(0.90-0.94) |
| 2005 | High SDI | 0.94(0.92-0.97) |
| 2006 | High SDI | 0.94(0.92-0.96) |
| 2007 | High SDI | 0.95(0.92-0.99) |
| 2008 | High SDI | 0.97(0.94-0.99) |
| 2009 | High SDI | 0.97(0.94-0.99) |
| 2010 | High SDI | 0.95(0.91-0.99) |
| 2011 | High SDI | 0.97(0.94-0.99) |
| 2012 | High SDI | 0.97(0.94-0.99) |
| 2013 | High SDI | 0.96(0.94-0.99) |
| 2014 | High SDI | 1.00(0.97-1.03) |
| 2015 | High SDI | 1.00(0.97-1.03) |
| 2016 | High SDI | 1.00(0.97-1.03) |
| 2017 | High SDI | 1.00(0.97-1.03) |
| 2018 | High SDI | 1.00(0.97-1.03) |
| 2019 | High SDI | 1.00(0.97-1.03) |
| 2020 | High SDI | 0.98(0.96-1.00) |
| 2021 | High SDI | 0.98(0.96-1.00) |
| 1990 | High-middle SDI | 1.73(1.69-1.78) |
| 1991 | High-middle SDI | 1.70(1.66-1.74) |
| 1992 | High-middle SDI | 1.72(1.69-1.75) |
| 1993 | High-middle SDI | 1.72(1.68-1.77) |
| 1994 | High-middle SDI | 1.70(1.67-1.73) |
| 1995 | High-middle SDI | 1.67(1.63-1.70) |
| 1996 | High-middle SDI | 1.67(1.63-1.72) |
| 1997 | High-middle SDI | 1.62(1.59-1.66) |
| 1998 | High-middle SDI | 1.64(1.61-1.68) |
| 1999 | High-middle SDI | 1.61(1.58-1.65) |
| 2000 | High-middle SDI | 1.56(1.51-1.61) |
| 2001 | High-middle SDI | 1.56(1.53-1.60) |
| 2002 | High-middle SDI | 1.47(1.44-1.51) |
| 2003 | High-middle SDI | 1.47(1.44-1.51) |
| 2004 | High-middle SDI | 1.44(1.41-1.47) |
| 2005 | High-middle SDI | 1.41(1.38-1.44) |
| 2006 | High-middle SDI | 1.42(1.39-1.45) |
| 2007 | High-middle SDI | 1.39(1.35-1.42) |
| 2008 | High-middle SDI | 1.35(1.32-1.38) |
| 2009 | High-middle SDI | 1.35(1.32-1.38) |
| 2010 | High-middle SDI | 1.32(1.28-1.35) |
| 2011 | High-middle SDI | 1.32(1.28-1.35) |
| 2012 | High-middle SDI | 1.27(1.24-1.30) |
| 2013 | High-middle SDI | 1.27(1.24-1.30) |
| 2014 | High-middle SDI | 1.23(1.20-1.26) |
| 2015 | High-middle SDI | 1.23(1.20-1.26) |
| 2016 | High-middle SDI | 1.23(1.20-1.26) |
| 2017 | High-middle SDI | 1.19(1.16-1.22) |
| 2018 | High-middle SDI | 1.20(1.17-1.23) |
| 2019 | High-middle SDI | 1.16(1.14-1.19) |
| 2020 | High-middle SDI | 1.18(1.15-1.21) |
| 2021 | High-middle SDI | 1.15(1.12-1.18) |
| 1990 | Middle SDI | 1.17(1.15-1.20) |
| 1991 | Middle SDI | 1.17(1.15-1.20) |
| 1992 | Middle SDI | 1.16(1.14-1.18) |
| 1993 | Middle SDI | 1.16(1.14-1.17) |
| 1994 | Middle SDI | 1.14(1.12-1.16) |
| 1995 | Middle SDI | 1.13(1.11-1.15) |
| 1996 | Middle SDI | 1.13(1.10-1.15) |
| 1997 | Middle SDI | 1.12(1.10-1.14) |
| 1998 | Middle SDI | 1.11(1.09-1.13) |
| 1999 | Middle SDI | 1.10(1.09-1.11) |
| 2000 | Middle SDI | 1.10(1.09-1.12) |
| 2001 | Middle SDI | 1.09(1.07-1.10) |
| 2002 | Middle SDI | 1.09(1.07-1.10) |
| 2003 | Middle SDI | 1.08(1.06-1.10) |
| 2004 | Middle SDI | 1.08(1.06-1.10) |
| 2005 | Middle SDI | 1.06(1.04-1.08) |
| 2006 | Middle SDI | 1.03(1.01-1.05) |
| 2007 | Middle SDI | 1.01(0.99-1.03) |
| 2008 | Middle SDI | 1.02(1.00-1.03) |
| 2009 | Middle SDI | 1.01(0.99-1.03) |
| 2010 | Middle SDI | 1.02(1.00-1.03) |
| 2011 | Middle SDI | 1.01(0.99-1.03) |
| 2012 | Middle SDI | 0.98(0.96-1.00) |
| 2013 | Middle SDI | 0.98(0.96-1.00) |
| 2014 | Middle SDI | 0.98(0.96-1.00) |
| 2015 | Middle SDI | 0.96(0.94-0.97) |
| 2016 | Middle SDI | 0.98(0.96-1.00) |
| 2017 | Middle SDI | 0.97(0.94-0.99) |
| 2018 | Middle SDI | 0.94(0.92-0.97) |
| 2019 | Middle SDI | 0.94(0.93-0.95) |
| 2020 | Middle SDI | 0.93(0.91-0.94) |
| 2021 | Middle SDI | 0.92(0.90-0.94) |
| 1990 | Low-middle SDI | 1.29(1.27-1.31) |
| 1991 | Low-middle SDI | 1.28(1.26-1.30) |
| 1992 | Low-middle SDI | 1.26(1.24-1.28) |
| 1993 | Low-middle SDI | 1.24(1.23-1.26) |
| 1994 | Low-middle SDI | 1.23(1.21-1.25) |
| 1995 | Low-middle SDI | 1.21(1.20-1.23) |
| 1996 | Low-middle SDI | 1.21(1.19-1.22) |
| 1997 | Low-middle SDI | 1.21(1.19-1.23) |
| 1998 | Low-middle SDI | 1.18(1.16-1.20) |
| 1999 | Low-middle SDI | 1.16(1.14-1.18) |
| 2000 | Low-middle SDI | 1.15(1.13-1.17) |
| 2001 | Low-middle SDI | 1.13(1.11-1.15) |
| 2002 | Low-middle SDI | 1.10(1.08-1.12) |
| 2003 | Low-middle SDI | 1.08(1.06-1.10) |
| 2004 | Low-middle SDI | 1.04(1.03-1.06) |
| 2005 | Low-middle SDI | 1.02(1.01-1.04) |
| 2006 | Low-middle SDI | 0.98(0.96-1.00) |
| 2007 | Low-middle SDI | 0.96(0.94-0.97) |
| 2008 | Low-middle SDI | 0.94(0.93-0.96) |
| 2009 | Low-middle SDI | 0.95(0.93-0.97) |
| 2010 | Low-middle SDI | 0.97(0.95-0.98) |
| 2011 | Low-middle SDI | 0.96(0.94-0.97) |
| 2012 | Low-middle SDI | 0.95(0.93-0.97) |
| 2013 | Low-middle SDI | 0.95(0.94-0.97) |
| 2014 | Low-middle SDI | 0.97(0.95-0.98) |
| 2015 | Low-middle SDI | 0.94(0.92-0.97) |
| 2016 | Low-middle SDI | 0.94(0.93-0.95) |
| 2017 | Low-middle SDI | 0.93(0.92-0.94) |
| 2018 | Low-middle SDI | 0.94(0.92-0.96) |
| 2019 | Low-middle SDI | 0.92(0.91-0.93) |
| 2020 | Low-middle SDI | 0.88(0.86-0.89) |
| 2021 | Low-middle SDI | 0.85(0.85-0.86) |
| 1990 | Low SDI | 1.20(1.17-1.23) |
| 1991 | Low SDI | 1.20(1.17-1.23) |
| 1992 | Low SDI | 1.19(1.17-1.22) |
| 1993 | Low SDI | 1.19(1.17-1.22) |
| 1994 | Low SDI | 1.11(1.08-1.14) |
| 1995 | Low SDI | 1.16(1.13-1.19) |
| 1996 | Low SDI | 1.16(1.14-1.19) |
| 1997 | Low SDI | 1.16(1.14-1.19) |
| 1998 | Low SDI | 1.17(1.14-1.19) |
| 1999 | Low SDI | 1.15(1.12-1.18) |
| 2000 | Low SDI | 1.13(1.10-1.16) |
| 2001 | Low SDI | 1.11(1.09-1.13) |
| 2002 | Low SDI | 1.08(1.06-1.11) |
| 2003 | Low SDI | 1.07(1.04-1.09) |
| 2004 | Low SDI | 1.06(1.03-1.08) |
| 2005 | Low SDI | 1.03(1.01-1.06) |
| 2006 | Low SDI | 1.01(0.98-1.04) |
| 2007 | Low SDI | 1.00(0.97-1.03) |
| 2008 | Low SDI | 0.98(0.96-1.01) |
| 2009 | Low SDI | 0.98(0.96-1.01) |
| 2010 | Low SDI | 0.99(0.97-1.02) |
| 2011 | Low SDI | 0.99(0.97-1.01) |
| 2012 | Low SDI | 0.97(0.94-0.99) |
| 2013 | Low SDI | 0.95(0.93-0.98) |
| 2014 | Low SDI | 0.94(0.92-0.97) |
| 2015 | Low SDI | 0.94(0.92-0.97) |
| 2016 | Low SDI | 0.93(0.90-0.95) |
| 2017 | Low SDI | 0.91(0.89-0.94) |
| 2018 | Low SDI | 0.90(0.88-0.92) |
| 2019 | Low SDI | 0.90(0.88-0.92) |
| 2020 | Low SDI | 0.87(0.85-0.89) |
| 2021 | Low SDI | 0.85(0.83-0.88) |

**TableS5**.Lifetime risks (%) of developing and dying PUD within selected age intervals by sex in 2021

| Sex | Brith to death | 40 to death | 50 to death | 60 to death | 70 to death |
| --- | --- | --- | --- | --- | --- |
| Developing |  |  |  |  |  |
| Both | 3.21(3.20,3.22) | 2.52(2.52,2.53) | 2.18(2.18,2.19) | 1.69(1.69,1.70) | 1.10(1.10,1.10) |
| Male | 3.21(3.20,3.22) | 2.49(2.49,2.50) | 2.10(2.09,2.10) | 1.55(1.54,1.55) | 0.93(0.92,0.93) |
| Female | 3.21(3.20,3.23) | 2.57(2.56,2.57) | 2.28(2.28,2.29) | 1.86(1.86,1.86) | 1.29(1.29,1.30) |
| Dying |  |  |  |  |  |
| Both | 0.35(0.34,0.35) | 0.33(0.33,0.33) | 0.32(0.32,0.32) | 0.29(0.29,0.29) | 0.23(0.23,0.23) |
| Male | 0.34(0.34,0.34) | 0.32(0.32,0.33) | 0.30(0.30,0.31) | 0.26(0.26,0.27) | 0.20(0.20,0.20) |
| Female | 0.35(0.35,0.36) | 0.34(0.34,0.34) | 0.33(0.33,0.34) | 0.31(0.31,0.31) | 0.26(0.26,0.27) |

**TableS6.**Lifetime and cumulative (ages 0-74) risks (%) of developing PUD by region worldwide in 2021

| Location | CR (95%CI) | Lifetime risk(95%CI) | Risk difference (95%CI) | p-value |
| --- | --- | --- | --- | --- |
| Global | 3.64 (3.62,3.66) | 3.21(3.20,3.22) | 0.43 (0.39,0.47)* | ＜0.05 |
| High SDI | 3.40 (3.37,3.44) | 3.85(3.81,3.88) | -0.45 (-0.55,-0.35)* | ＜0.05 |
| High-middle SDI | 3.05 (3.01,3.08) | 2.89(2.87,2.92) | 0.16 (0.08,0.24)* | ＜0.05 |
| Middle SDI | 3.54 (3.51,3.56) | 3.14(3.13,3.15) | 0.40 (0.35,0.45)* | ＜0.05 |
| Low-middle SDI | 4.26 (4.22,4.30) | 3.20(3.18,3.21) | 1.06 (0.98,1.14)* | ＜0.05 |
| Low SDI | 4.12 (4.06,4.18) | 2.75(2.74,2.77) | 1.37 (1.25,1.49)* | ＜0.05 |
| Regional |  |  |  |  |
| Southern Latin America | 1.45 (1.35,1.55) | 1.48(1.44,1.53) | -0.03 (-0.24,0.18) | ＞0.05 |
| Western Europe | 1.46 (1.42,1.50) | 1.86(1.83,1.89) | -0.40 (-0.50,-0.30)* | ＜0.05 |
| High-income North America | 4.55 (4.49,4.62) | 4.76(4.71,4.80) | -0.21 (-0.36,-0.06)* | ＜0.05 |
| Australasia | 1.42 (1.28,1.56) | 2.00(1.89,2.11) | -0.58 (-0.93,-0.23)* | ＜0.05 |
| High-income Asia Pacific | 3.66 (3.57,3.75) | 4.78(4.57,5.00) | -1.12 (-1.58,-0.66)* | ＜0.05 |
| Caribbean | 2.51 (2.34,2.68) | 2.11(2.02,2.20) | 0.40 (0.02,0.78)* | ＜0.05 |
| Central Latin America | 1.79 (1.73,1.86) | 1.66(1.64,1.68) | 0.13 (-0.00,0.26) | ＞0.05 |
| Tropical Latin America | 1.92 (1.85,1.99) | 1.81(1.79,1.83) | 0.11 (-0.03,0.25) | ＞0.05 |
| Andean Latin America | 2.22 (2.07,2.37) | 1.89(1.84,1.94) | 0.33 (0.02,0.64)* | ＜0.05 |
| Central Sub-Saharan Africa | 3.64 (3.45,3.82) | 2.30(2.25,2.34) | 1.34 (0.97,1.71)* | ＜0.05 |
| Eastern Sub-Saharan Africa | 3.04 (2.95,3.14) | 1.97(1.95,1.99) | 1.07 (0.88,1.26)* | ＜0.05 |
| Southern Sub-Saharan Africa | 3.24 (3.07,3.42) | 1.84(1.81,1.87) | 1.40 (1.05,1.75)* | ＜0.05 |
| Western Sub-Saharan Africa | 4.37 (4.27,4.47) | 2.94(2.91,2.98) | 1.43 (1.22,1.64)* | ＜0.05 |
| North Africa and Middle East | 2.85 (2.80,2.91) | 2.41(2.39,2.44) | 0.44 (0.32,0.56)* | ＜0.05 |
| South Asia | 4.25 (4.21,4.29) | 3.30(3.28,3.32) | 0.95 (0.86,1.04)* | ＜0.05 |
| East Asia | 3.96 (3.92,3.99) | 3.89(3.87,3.92) | 0.07 (-0.01,0.15)* | ＜0.05 |
| Southeast Asia | 3.78 (3.72,3.84) | 3.16(3.13,3.19) | 0.62 (0.49,0.75)* | ＜0.05 |
| Oceania | 6.68 (6.02,7.34) | 4.60(4.35,4.86) | 2.08 (0.69,3.47)* | ＜0.05 |
| Central Asia | 3.46 (3.30,3.62) | 2.75(2.68,2.81) | 0.71 (0.37,1.05)* | ＜0.05 |
| Eastern Europe | 3.92 (3.83,4.01) | 3.08(3.04,3.13) | 0.84 (0.64,1.04)* | ＜0.05 |
| Central Europe | 3.99 (3.88,4.11) | 3.59(3.49,3.69) | 0.40 (0.10,0.70)* | ＜0.05 |

CR=Cumulative Risk


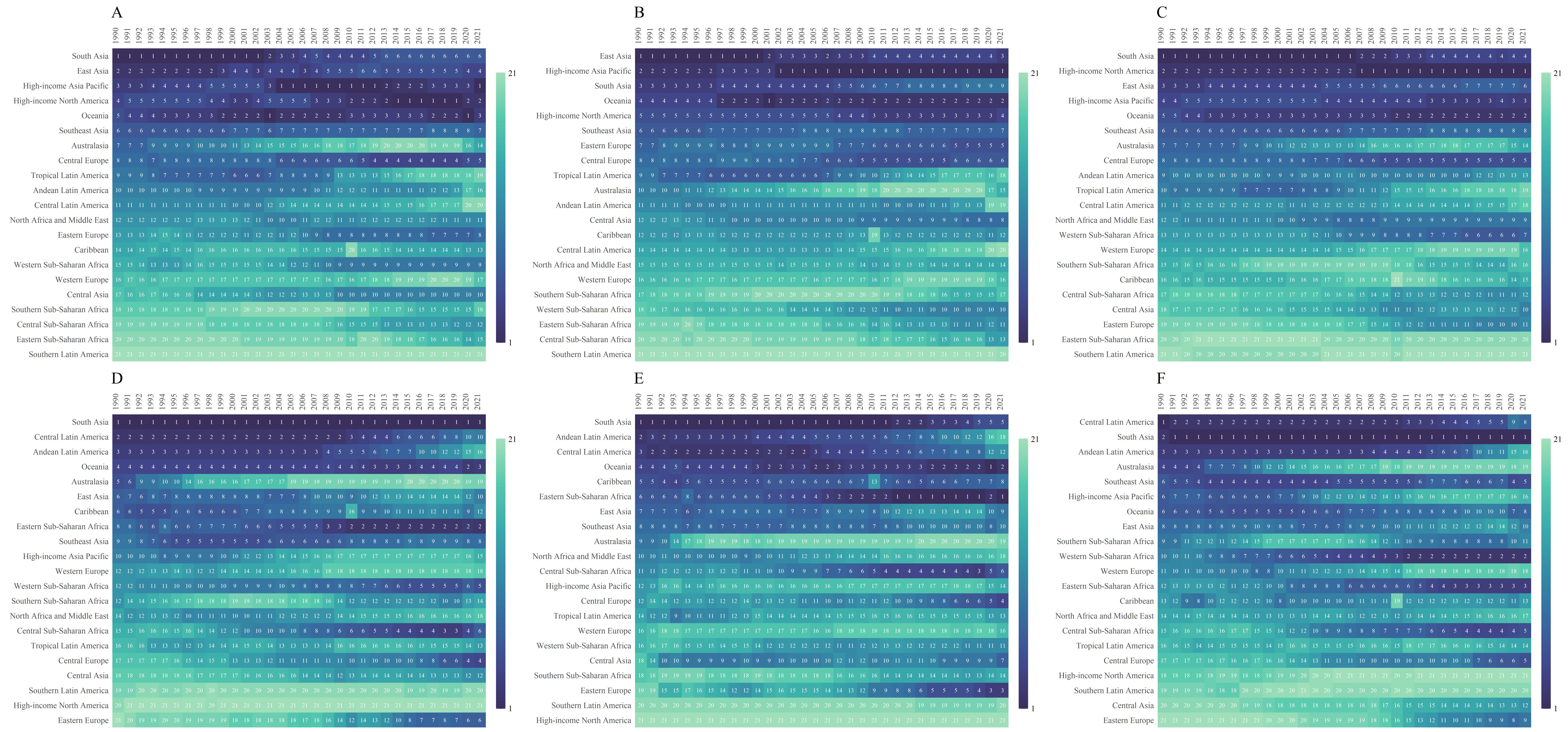


**Fig.S****1**.Heatmap of the lifetime risk of developing and dying from PUD for the whole population,males and females from 1990 to 2021. **A** Lifetime risk of developing PUD in the whole population. **B** Lifetime risk of developing PUD in males. **C** Lifetime risk of developing PUD in females. **D** Lifetime risk of dying PUD in the whole population. **E** Lifetime risk of dying PUD in males. **F** Lifetime risk of dying PUD in females.


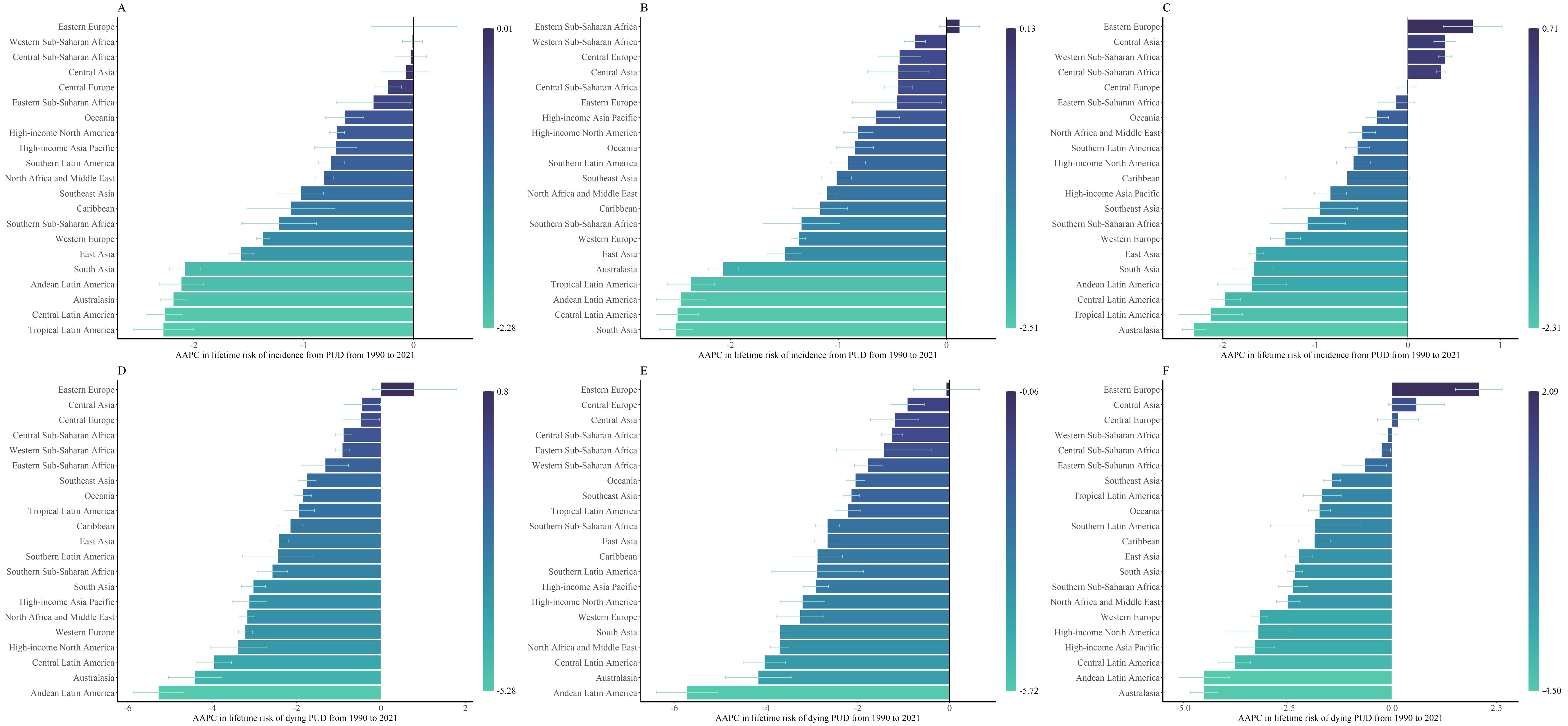


**Fig.S****2**.AAPC in lifetime risk of developing and dying from PUD for the whole population,males and females from 1990 to 2021 **A** AAPC in lifetime risk of developing from PUD in the whole population. **B** AAPC in lifetime risk of developing from PUD in males. **C** AAPC in lifetime risk of developing from PUD in females. **D** AAPC in lifetime risk of dying from PUD in the whole population. **E** AAPC in lifetime risk of dying from PUD in males. **F** AAPC in lifetime risk of dying in females.


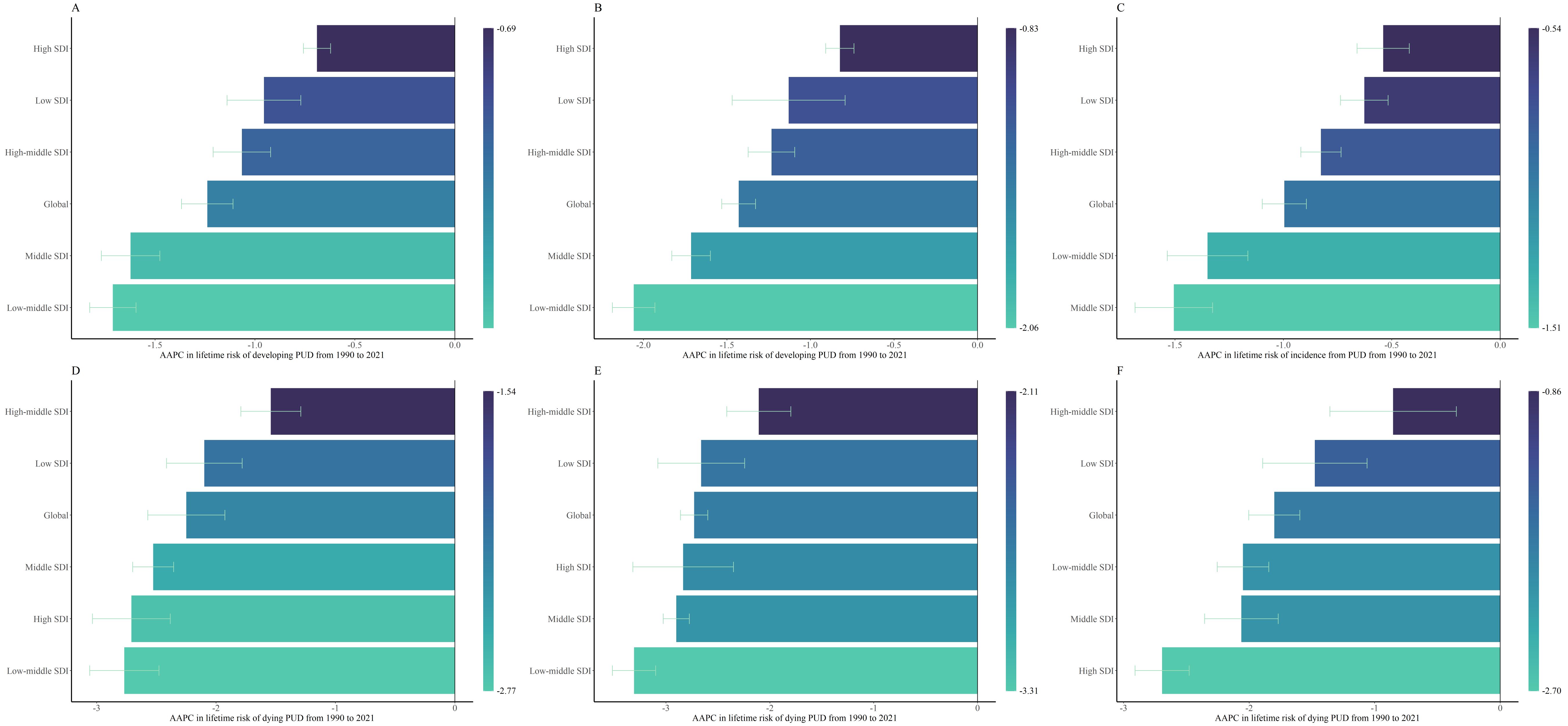


**Fig.S3**.AAPC in lifetime risk of developing and dying from PUD by SDI for the whole population,males and females from 1990 to 2021. **A** AAPC in lifetime risk of developing from PUD in the whole population. **B** AAPC in lifetime risk of developing from PUD in males. **C** AAPC in lifetime risk of developing from PUD in females. **D** AAPC in lifetime risk of dying from PUD in the whole population. **E** AAPC in lifetime risk of dying from PUD in males. **F** AAPC in lifetime risk of dying from PUD in females.

**
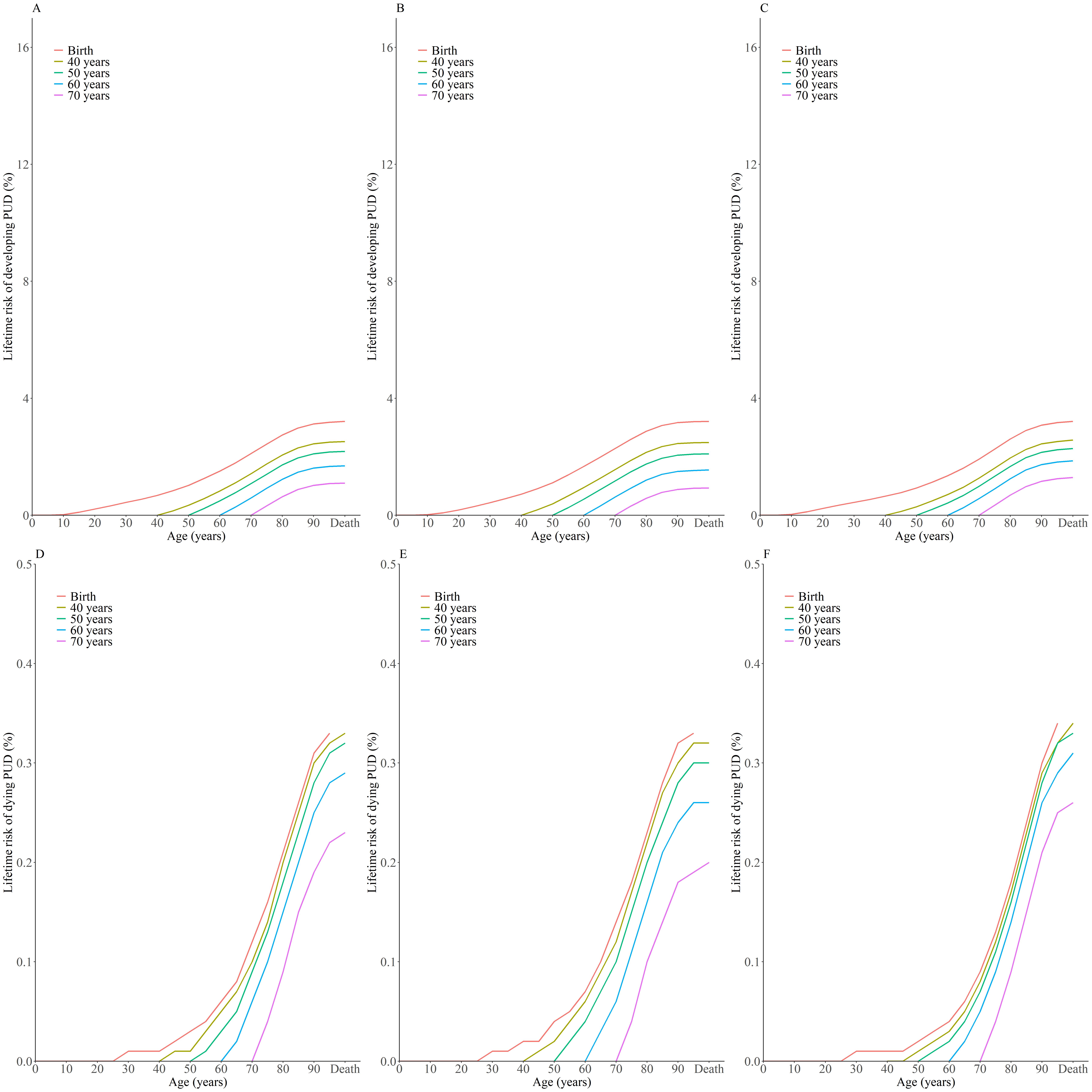

Fig.S4.** Lifetime risks of developing and dying from PUD for the whole population,males and females by age at diagnosis in 2021. **A** Lifetime risk of developing PUD in the whole population. **B** Lifetime risk of developing PUD in males. **C** Lifetime risk of developing PUD in females. **D** Lifetime risk of dying PUD in the whole population. **E** Lifetime risk of dying PUD in males. **F** Lifetime risk of dying PUD in females.


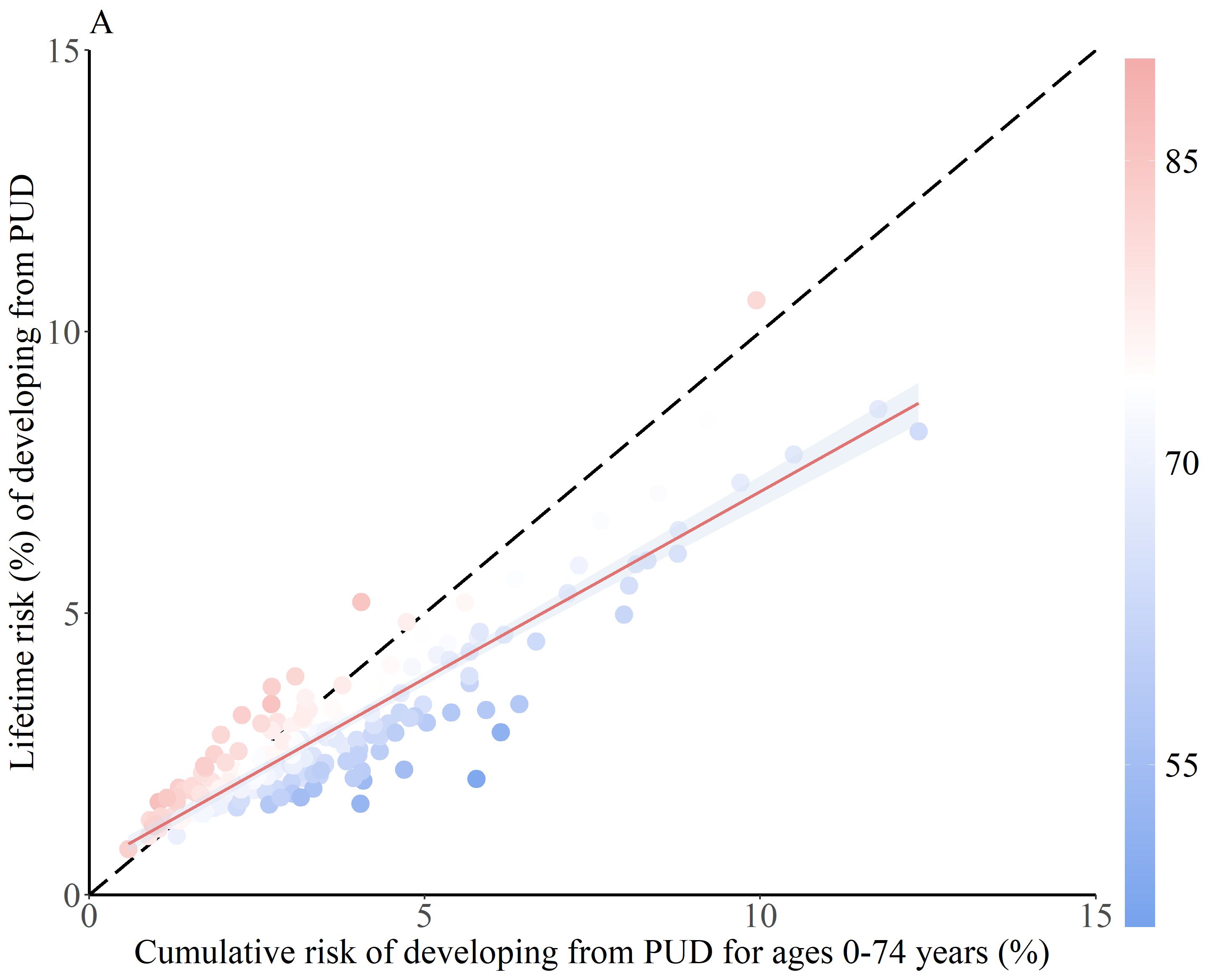


**Fig.S5**.Correlation between lifetime risk of developing PUD and traditional cumulative risk measure (ages 0-74 years) with both sexes combined.The red line indicates the fitted risks using quadratic regression with a 95% confidence interval; the short dash black line indicates the reference line where the risk estimations of two measures are equal; the color circles indicate the risks in different countries/regions with different life expectancy.
